# Induction immunochemotherapy followed by concurrent chemoradiotherapy improves survival in unresectable esophageal cancer: a systematic review, meta-analysis, and network meta-analysis

**DOI:** 10.3389/fimmu.2026.1767380

**Published:** 2026-05-12

**Authors:** Xuefei Fan, Xin Liu, Song Mi, Yunxin Yang, Chenggong Li, Jiandong Zhang, Pingping Hu

**Affiliations:** 1 Department of Oncology, The First Affiliated Hospital of Shandong First Medical University & Shandong Provincial Qianfoshan Hospital, Shandong Lung Cancer Institute, Jinan, China; 2 Department of Oncology, Yuncheng Yanhu People’s Hospital, Yuncheng, Shanxi, China; 3 Cheeloo College of Medicine, Shandong University, Jinan, China

**Keywords:** concurrent chemoradiotherapy, consolidation immunotherapy, esophageal cancer, induction immunochemotherapy, meta-analysis

## Abstract

**Background:**

Concurrent chemoradiotherapy (CCRT) remains the standard treatment for unresectable esophageal cancer (EC). However, the clinical benefits of combining immunotherapy with CCRT for unresectable EC remain controversial. Therefore, we conducted a systematic review and meta-analysis to evaluate the potential benefits of different immunotherapy strategies added to CCRT in patients with unresectable EC.

**Methods:**

We employed single-arm, pairwise and network meta-analysis (NMA) methods to analyze the overall survival (OS), progression-free survival (PFS), objective response rate (ORR) and safety of several combined treatment strategies based on CCRT.

**Results:**

30 single-arm trials and 39 controlled trials involving a total of 9648 participants were included. Compared with CCRT alone, both induction immunochemotherapy plus CCRT (ICT-CCRT) and CCRT plus consolidation immunotherapy (CCRT-IO) significantly improved OS in pairwise meta-analyses (HR 0.52, 95% CI 0.39–0.71, I²= 0.0%; HR 0.77, 95% CI 0.65–0.90, I²= 0.0%, respectively). In the NMA, only ICT-CCRT showed a significant OS benefit (HR 0.75, 95% CI 0.64–0.88), whereas CCRT-IO did not. For PFS, CCRT-IO consistently prolonged PFS compared with CCRT alone in both pairwise meta-analyses (HR 0.74, 95% CI 0.64-0.85, I²=0.0%) and NMA (HR 0.78, 95% CI 0.76–0.99). In contrast, ICT-CCRT demonstrated a significant PFS benefit only in the NMA (HR 0.84, 95% CI 0.71–0.99), but not in the pairwise analysis. Notably, ICT-CCRT ranked first in the NMA for both OS and PFS.

**Conclusions:**

In conclusion, compared with CCRT alone, ICT-CCRT significantly improves OS and shows a potential PFS benefit. CCRT-IO improves OS only in pairwise analyses but consistently improves PFS across analyses. Currently, sufficient randomized controlled trials are lacking to validate the efficacy and determine the optimal timing and sequence.

**Systematic review registration:**

https://www.crd.york.ac.uk/prospero/, identifier CRD420261364669.

## 1 Introduction

Esophageal carcinoma (EC) is one of the most common cancers worldwide and the seventh leading cause of cancer-related death (1), with 30%−40% of cases diagnosed initially as unresectable locally advanced disease (2). The disease shows marked geographic variation, with predominantly squamous cell carcinoma in East Asia and adenocarcinoma in Western countries (3). Since 1999, definitive concurrent chemoradiotherapy (dCCRT) has been the standard treatment for medically inoperable or locally advanced EC, based on the RTOG 85–01 trial (4–6). However, this approach remains associated with significant limitations, including local recurrence rates of 40%−60% and 5-year overall survival (OS) rates below 20% (2). Therefore, there is a need for a more effective method to further improve the survival rate of EC patients who undergo concurrent chemoradiotherapy (CCRT).

In recent years, combining immunotherapy with CCRT has become a new approach for treating locally advanced EC, potentially showing synergistic effects and improved efficacy (7). Several clinical studies have shown promising short-term efficacy and acceptable safety with neoadjuvant immunotherapy plus chemotherapy in patients with resectable locally advanced esophageal squamous cell carcinoma (ESCC) (8–11). In unresectable EC, several combined treatment strategies—such as induction immunochemotherapy plus CCRT (ICT-CCRT), CCRT plus concurrent immunotherapy (CCRT-cIO), CCRT plus consolidation immunotherapy (CCRT-IO), induction chemotherapy plus CCRT (CT-CCRT), and CCRT plus consolidation chemotherapy (CCRT-CT) —have been explored to improve outcomes. Preliminary data from small studies show promising efficacy and acceptable safety of CCRT-cIO in unresectable locally advanced EC (12, 13). Additionally, evidence has suggested that ICT-CCRT may significantly improve OS and progression-free survival (PFS) (14, 15). However, the optimal treatment mode for unresectable EC remains unclear and needs to be determined by further research.

Owing to the limited number of publications, inconsistent findings, and the lack of prospective randomized controlled studies and direct comparisons between several treatment regimens, the value of CCRT-based combination therapies in EC patients remains controversial. Therefore, using available published data, we conducted a single-arm, pairwise, and network meta-analysis to evaluate the comparative efficacy and safety of CCRT-based combination treatment strategies, which is critical for clinicians when making optimal treatment decision for patients with unresectable locally advanced EC.

## 2 Methods

### 2.1 Search strategy

We searched the PubMed, Embase, Cochrane Library, Web of Science, and ClinicalTrials.gov databases for clinical studies from database inception to March 31, 2026. Search keywords included “Esophageal Neoplasms” and “induction or neoadjuvant therapy” and “consolidation therapy” and “immune checkpoint inhibitors (ICIs)” and “programmed cell death protein 1 (PD-1)” or “programmed cell death ligand 1 (PD-L1)” or “cytotoxic T-lymphocyte-associated protein 4 (CTLA-4)”. We also reviewed abstracts and presentations from major international conferences--including American Society of Clinical Oncology Annual Meeting (ASCO), European Society for Medical Oncology Congress (ESMO), and American Society for Radiation Oncology Annual Meeting (ASTRO) from 2023 to 2025, and we checked reference lists of published reviews and meta-analyses to ensure no studies were overlooked. The detailed literature search process is presented in **Supplementary Table 1**.

### 2.2 Inclusion and exclusion criteria

Eligibility criteria included:(1) published or unpublished clinical trials or observational studies; unpublished studies were defined as those identified solely through clinical trial registries (e.g., ClinicalTrials.gov) or conference abstracts; (2) adult patients with histologically confirmed, unresectable, locally advanced EC who had not undergone prior surgical resection; (3) studies evaluating CCRT or radiotherapy as definitive local treatment, with eligible regimens including additional systemic therapy, such as induction or consolidation chemotherapy, or immunotherapy given as induction immunochemotherapy, concurrent immunotherapy, or consolidation immunotherapy; (4) studies that reported at least one of the outcomes mentioned below.

Exclusion criteria included: (1) non−English language publications or studies with insufficiently reported data from which relevant outcome measures could not be extracted; (2) studies in which patients had undergone surgical resection of EC, or had distant metastases, recurrent EC, or non-esophageal primary tumors; (3) multiple reports derived from the same patient cohort; in such cases, only the most recent or most complete dataset was retained for analysis.

### 2.3 Data collection

Two researchers independently extracted the following items from each included study: first author, year of publication or conference presentation, country and region, research type, sample size, number in the experimental group, number in the control group, follow-up time, pathological type, clinical stage, treatment regimen, outcomes (hazard ratios (HRs) and their corresponding 95% confidence intervals (CIs) for PFS and OS, the 1-year and 2-year PFS rates and OS rates, objective response rate (ORR) and severe-grade adverse events(AEs)). The extracted data were cross-checked, and discrepancies were resolved by consensus through group discussion or arbitration by a third senior investigator. Missing data were supplemented by contacting the corresponding author or derived from the original text. However, if sufficient data required for the meta-analysis could not be obtained after attempts to contact the authors, the study was excluded.

### 2.4 Data analysis

The data analysis was conducted using Stata software (version 18.0) and R software (version 4.4.2), in which Stata software was utilized for single-group (metaprop function) and paired (metan function) meta-analysis, while the gemtc package of R software was employed for network meta-analysis (NMA). The analyzed outcomes included OS, PFS, ORR, and grade ≥3 AEs. The risk of bias in the included studies was assessed using two tools: the Methodological Index for Non-Randomized Studies (MINORS) for single-arm and non-randomized observational trials (16), and the Cochrane risk of bias tool for randomized controlled trials (17). The work has been reported in accordance with the Preferred Reporting Items for Systematic Reviews and Meta-Analyses (PRISMA) guidelines (18). The review protocol was registered in PROSPERO (identifier CRD420261364669) and can be accessed at https://www.crd.york.ac.uk/PROSPERO/view/CRD420261364669.

The single-arm meta-analysis was conducted to calculate pooled estimates of clinical outcomes including 1-year and 2-year OS rates, 1-year and 2-year PFS rates, ORR and incidence of severe-grade AEs. The pairwise meta-analysis was conducted for head-to-head comparisons involving two or more controlled trials. HRs and their corresponding 95% CIs were used as the primary metrics for assessing survival outcomes (PFS and OS). When possible, HRs and corresponding 95% CIs were obtained directly from the studies. If exact HR values were not reported in the original study, they were estimated from the survival curve (19). For both the single-arm and pairwise meta-analyses, heterogeneity was evaluated using the I² statistic and the Q-test (20). When statistical heterogeneity was substantial (I²>50% or Q-test p<0·10), the random-effects model was employed; otherwise, the fixed-effect model was adopted. For analyses including fewer than five studies, the random-effects model was used regardless of heterogeneity estimates, owing to the low power of I² in small meta-analyses. When the number of included studies was sufficient (more than ten), subgroup analyses were conducted to explore potential sources of heterogeneity.

We performed a NMA utilizing the Markov chain Monte Carlo simulation technique to compare any two treatment strategies by integrating both direct and indirect evidence simultaneously (21). In addition, this approach allowed for the comparison of pairs of interventions that were not directly evaluated in the reported trials. This comprehensive comparison of all interventions in a single analysis also estimated their relative efficacy ranking for a specific outcome (22, 23). We conducted the NMA within the Bayesian framework using a random-effects model and determined whether the residual deviance approximated the number of data points by posterior mean (24). Four distinct chains were run with 50,000 iterations, discarding 20,000 initial burn-in iterations per chain. Summary estimate statistics were reported as HRs for the corresponding outcomes, along with their 95% CIs. We assessed the transitivity by examining the baseline characteristics of the included studies (age, gender, clinical stage, pathological type, treatment protocols) (25). Global inconsistency was assessed by comparing the fit of consistency and inconsistency models. Local inconsistency between direct and indirect results was assessed by using the node-splitting method. The probability of each treatment being the most effective was calculated using the surface under the cumulative ranking curve (SUCRA). For each outcome, the greater the SUCRA value, the better the rank of a certain therapy among the various treatments.

We also conducted sensitivity analyses on the primary outcomes using both fixed-effect and random-effects models to assess the robustness of the results. Outliers were addressed by removing studies whose point estimate of the effect size fell outside the confidence interval of the pooled effect size (26). Funnel plots were employed to assess potential publication bias. Egger’s regression test was conducted to evaluate the asymmetry of the funnel plot. A P value of less than 0.05 was considered indicative of publication bias.

## 3 Results

**Figure 1:**
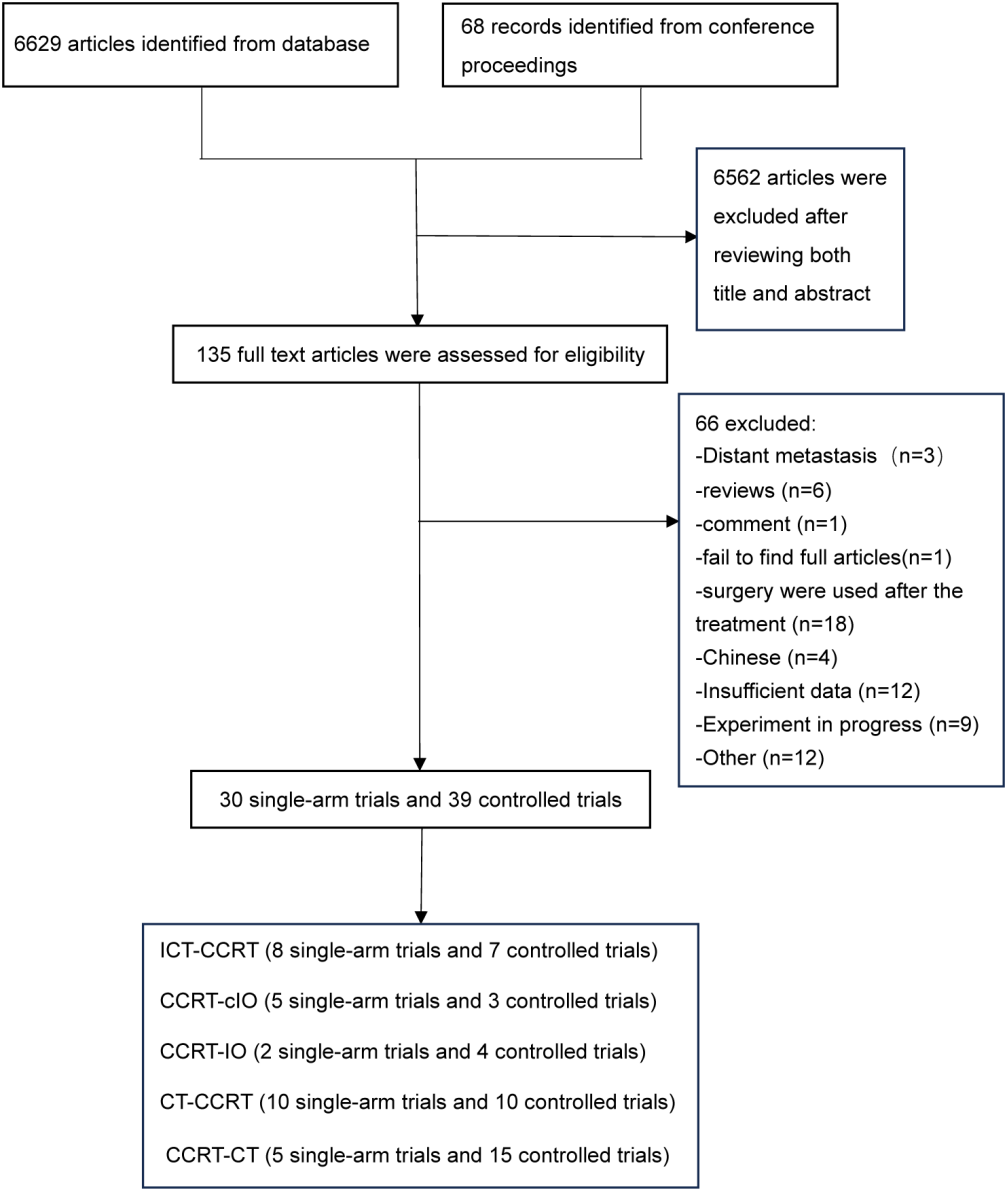
Flow chart of the study selection process. CCRT, concurrent chemoradiotherapy; ICT-CCRT, induction immunochemotherapy plus CCRT; CCRT-cIO, CCRT plus concurrent immunotherapy; CCRT-IO, CCRT plus consolidation immunotherapy; CT-CCRT, induction chemotherapy plus CCRT; CCRT-CT, CCRT plus consolidation chemotherapy.

6629 records through database searches and an additional 68 studies from conference proceedings were identified. Among these, 135 full-text articles were evaluated for eligibility, and 66 articles that did not meet the inclusion criteria were excluded—as summarized in **Figure 1**. Finally, 69 articles were included in our meta-analysis, comprising 30 single-arm trials (12, 13, 27–54) and 39 controlled studies (5, 14, 15, 55–90), which together included a total of 9648 unresectable locally advanced EC patients. These trials evaluated six treatment strategies for patients with unresectable locally advanced EC: ICT-CCRT (14, 15, 27–32, 55–57, 86, 87), CCRT-cIO (12, 13, 33–35, 58, 59, 85), CCRT-IO (36, 37, 60, 61, 88, 89), CT-CCRT (38–47, 62–70, 90), CCRT-CT (5, 48–52, 71–84), and CCRT (14, 15, 58, 59, 61–68, 70, 71, 73–82). Through sensitivity analysis, we excluded the study by Xie et al. from the pairwise and network meta-analysis. The basic characteristics of the included studies are detailed in the **Supplementary Table 2**. Among the 69 included studies, 27 were prospective studies, and the remainder were retrospective studies. The majority of patients included in the studies had ESCC, and most were stage III-IV according to the American Joint Committee on Cancer TNM staging system. The chemotherapy regimens in most trials were primarily based on taxanes and platinum drugs, while the radiation doses administered in the radiotherapy protocols ranged from 50 to 60 Gy across most trials. Based on these baseline characteristics, the transitivity assumption was considered satisfied. According to the RoB 2 tool, six of the eight randomized controlled trials were assessed as having a low risk of bias, while the remaining two were judged to raise some concerns. For the non-randomized studies, the MINORS scores indicated moderate to high methodological quality for the majority of included trials, with only one study rated as low quality. The specific quality assessment results are presented in **Supplementary Figure 1** and the last column of **Supplementary Table 2**.

### 3.1 OS benefit with the combination of immunotherapy with CCRT

In the single-arm analysis, 62 studies contributed 1-year OS rate and 57 contributed 2-year OS rate. Summarizing the data of single-arm and the experimental group of controlled trials, ICT-CCRT achieved the most favorable estimates (1-year OS: 86.1%, 95% CI 82.2-89.6, I²=70.0%; 2-year OS: 64.9%, 95% CI 58.0-71.6, I²=77.9%). The other immunotherapy−combined regimens also yielded numerically higher OS estimates compared with chemotherapy-combined regimens and CCRT alone. Detailed estimates for all treatment strategies are summarized in **Table 1**. Given the substantial heterogeneity observed in the ICT−CCRT group, a subgroup analysis by research type (retrospective versus prospective) was performed, with results shown in **Supplementary Figure 2**.

**Table 1:** Efficacy of the 6 treatment regimens in single meta-analysis.

Treatment regimen (Studies)	NO. of study	NO. of patient	The pooled 1-year OS rate(95%CI)	NO. of study	NO. of patients	The pooled 2-year OS rate (95%CI)	NO. of study	NO. of patients	The pooled 1-year PFS rate (95%CI)	NO. of study	NO. of patients	The pooled 2-year PFS rate (95%CI)	NO. of study	NO. ofpatients	The pooled ORR (95%CI)
ICT-CCRT	16	1242	86.1% (82.2-89.6)	12	924	64.9% (58.0-71.6)	15	1151	70.5% (66.2-74.7)	11	883	49.3% (41.8-56.8)	7	441	88.4% (78.9-95.5)
CCRT-cIO	9	530	82.6% (79.1-85.8)	6	429	63.2% (54.2-71.8)	9	530	65.3% (61.0-69.3)	6	429	46.1% (32.9-59.5)	7	388	70.4% (61.8-78.4)
CCRT-IO	6	741	82.7% (76.4-88.2)	5	701	62.4% (54.3-70.1)	6	741	64.7% (56.1-72.9)	5	701	49.1% (45.3-52.8)	2	93	69.0% (59.0-78.1)
CT-CCRT	19	1670	73.8% (67.9-79.2)	19	1664	48.4% (42.3-54.6)	8	804	66.1% (56.6-75.1)	8	804	42.0% (32.9-51.3)	10	439	71.9% (63.0-80.1)
CCRT-CT	19	1988	79.0% (73.8-83.9)	20	2022	56.9% (51.6-62.1)	11	1288	70.0% (63.5-76.1)	11	1288	52.1% (47.7-56.5)	5	361	84.9% (69.1-95.9)
CCRT	28	3287	71.8% (67.0-76.4)	26	3174	49.1% (44.5-53.7)	20	2586	58.8% (54.4-63.0)	18	2473	40.3% (35.9-44.7)	8	510	61.0% (48.2-73.1)
Included studies	(5, 12–15, 27–37, 39–43, 45, 46, 49–51, 53, 54, 56–68, 70–90)			(5, 12, 14, 15, 27, 29, 31–33, 35–38, 40–43, 45, 46, 48–51, 53, 54, 56, 57, 59–68, 70–85, 87–90)			(12–15, 27–37, 40, 49, 50, 53–61, 65–68, 70, 71, 73, 74, 78–81, 83–86, 88, 89)			(12, 14, 15, 27, 29, 31–33, 35–37, 40, 49, 50, 53, 54, 56, 57, 59–61, 65–68, 70, 71, 73, 74, 78–81, 83–85, 88, 89)			(12, 13, 15, 28, 30–35, 42, 43, 46, 49, 51, 53, 55, 58, 64, 68, 69, 74, 77, 83, 85, 90)		

The data from single-arm and the experimental group of controlled trials. CI, confidence interval; OS, overall survival; PFS, progression-free survival; ORR, objective response rate; CCRT, concurrent chemoradiotherapy; ICT-CCRT, induction immunochemotherapy plus CCRT; CCRT-cIO, CCRT plus concurrent immunotherapy; CCRT-IO, CCRT plus consolidation immunotherapy; CT-CCRT, induction chemotherapy plus CCRT; CCRT-CT, CCRT plus consolidation chemotherapy.

**Figure 2:**
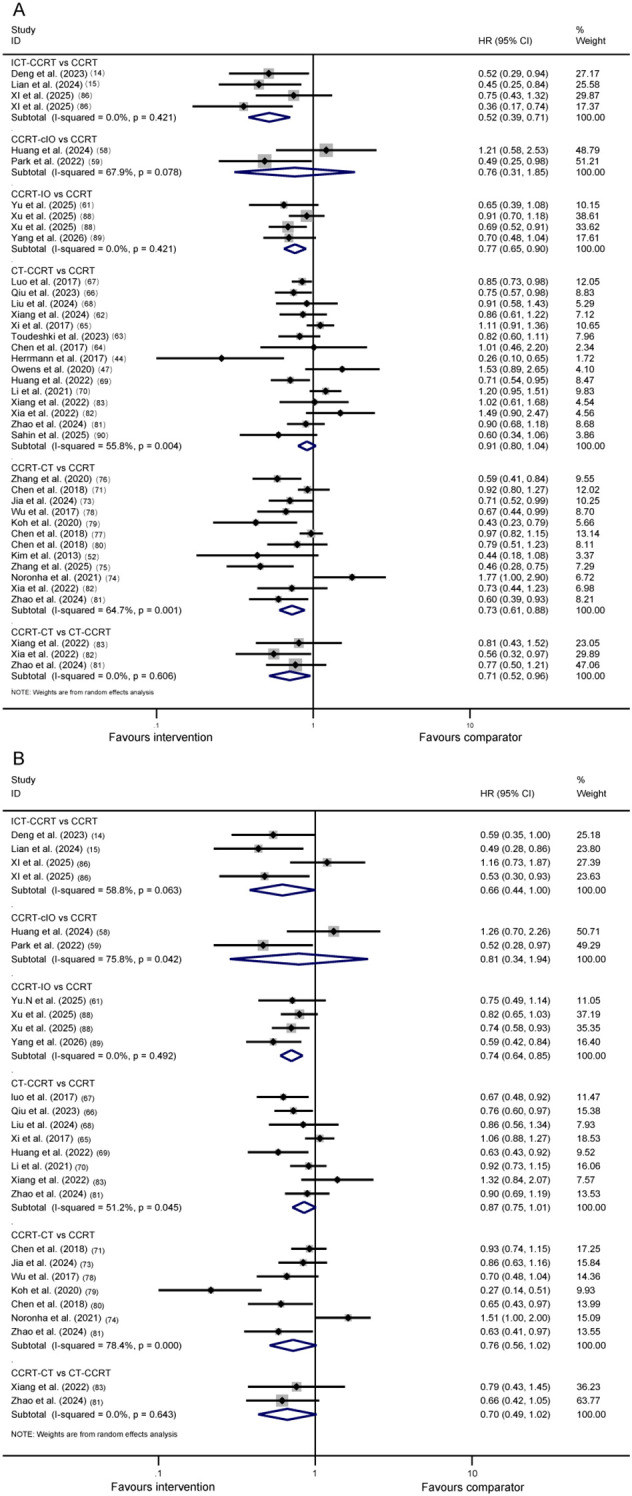
Pooled OS and PFS of each head-to-head comparison in pairwise meta-analysis. Pooled HRs for **(A)** OS and **(B)** PFS with their corresponding 95% CIs. OS, overall survival; PFS, progression-free survival; HR, hazard ratio; CI, confidence interval; CCRT, concurrent chemoradiotherapy; ICT-CCRT, induction immunochemotherapy plus CCRT; CCRT-cIO, CCRT plus concurrent immunotherapy; CCRT-IO, CCRT plus consolidation immunotherapy; CT-CCRT, induction chemotherapy plus CCRT; CCRT-CT, CCRT plus consolidation chemotherapy.

In the pairwise meta-analyses, 33 studies were included to compare OS across treatment strategies, including ICT-CCRT versus CCRT, CCRT-IO versus CCRT, CCRT-cIO versus CCRT, CT-CCRT versus CCRT, CCRT-CT versus CCRT, and CCRT-CT versus CT-CCRT. Pooled OS of each head-to-head comparison in **Figure 2A**. Compared with CCRT alone, both ICT-CCRT (HR 0.52, 95% CI 0.39–0.71, I² = 0.0%) and CCRT-IO (HR 0.77, 95% CI 0.65–0.90, I² = 0.0%) significantly prolonged OS. In addition, CCRT-CT had superior OS to CCRT (HR 0.73, 95% CI 0.61-0.88, I²=64.7%) and CT-CCRT (HR 0.71, 95%CI 0.52-0.96, I²=0.0%). No statistically significant differences were observed among the remaining pairwise comparisons. Subgroup analyses showed that in the CCRT−CT versus CCRT comparison, intravenous consolidation chemotherapy improved OS (HR 0.70, 95% CI 0.59–0.83, I² = 55.7%), while oral regimens were limited to one study (HR 1.77, 95% CI 1.04–3.11). For CT−CCRT versus CCRT, OS benefit was observed in studies with predominantly squamous cell carcinoma (HR 0.85, 95% CI 0.75–0.96, I² = 14.6%), but not in the two adenocarcinoma−predominant studies (**Supplementary Figure 3**).

**Figure 3:**
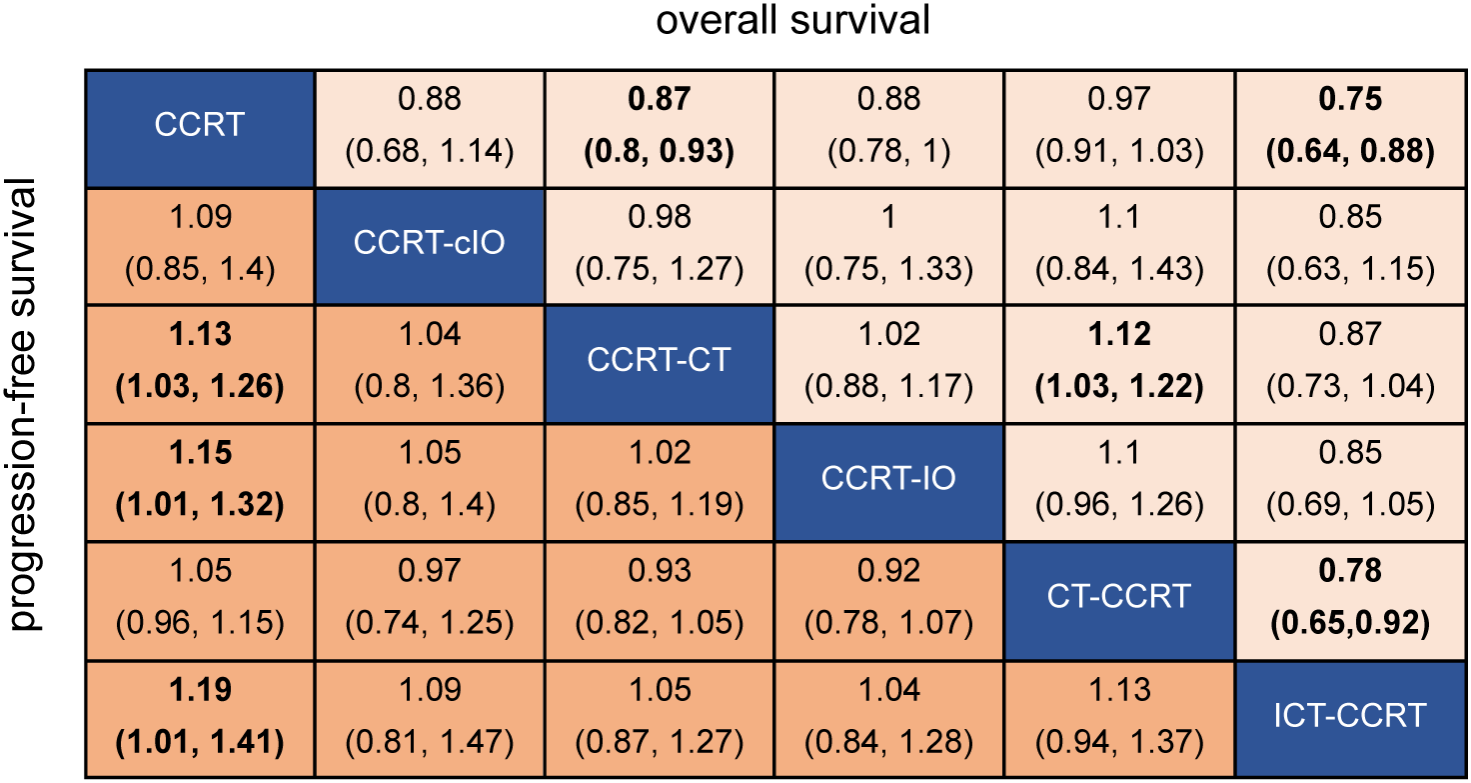
Efficacy of the 6 treatment regimens in network meta-analysis. Data are pooled HR (95% CI). Bold data indicate a significant difference. HR, hazard ratio; CI, confidence interval; CCRT, concurrent chemoradiotherapy; ICT-CCRT, induction immunochemotherapy plus CCRT; CCRT-cIO, CCRT plus concurrent immunotherapy; CCRT-IO, CCRT plus consolidation immunotherapy; CT-CCRT, induction chemotherapy plus CCRT; CCRT-CT, CCRT plus consolidation chemotherapy.

**Figure 4:**
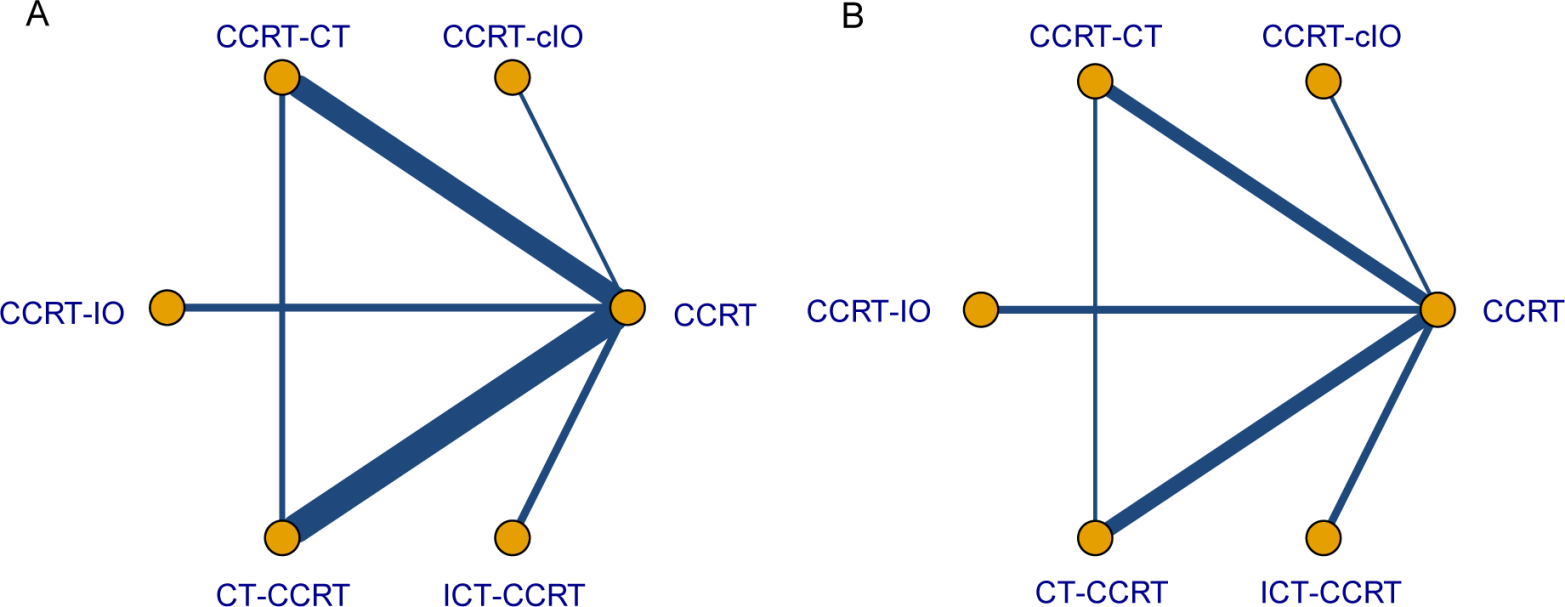
Eligible comparisons for each outcome in the network meta-analysis. Network plots illustrating the direct and indirect comparisons for **(A)** OS and **(B)** PFS. Circular nodes represent treatment strategies. Lines represent the direct comparisons, with thicknesses proportional to the number of involved studies. OS, overall survival; PFS, progression-free survival; CCRT, concurrent chemoradiotherapy; ICT-CCRT, induction immunochemotherapy plus CCRT; CCRT-cIO, CCRT plus concurrent immunotherapy; CCRT-IO, CCRT plus consolidation immunotherapy; CT-CCRT, induction chemotherapy plus CCRT; CCRT-CT, CCRT plus consolidation chemotherapy.

In the NMA, a total of 28 studies were included. Consistent with the pairwise meta-analysis, ICT−CCRT was associated with improved OS compared with CCRT alone (HR 0.75, 95% CI 0.64–0.88) and CT−CCRT (HR 0.78, 95% CI 0.65–0.92). Similarly, CCRT−CT demonstrated better OS than both CCRT alone (HR 0.87, 95% CI 0.80–0.93) and CT−CCRT (HR 0.89, 95% CI 0.82–0.97). In contrast, the comparison between CCRT−IO and CCRT alone did not reach statistical significance (HR 0.88, 95% CI 0.78–1.00). No significant differences were observed among the remaining treatment strategies (**Figure 3**). The network diagram of the evaluated regimens is presented in **Figure 4A**, and the ranking probabilities for OS are shown in **Supplementary Figure 4A**. Among the included strategies, ICT−CCRT had the highest probability of ranking first (0.95). Global and local assessments of inconsistency supported the assumption of network consistency. Details are provided in **Supplementary Table 3**; **Supplementary Figure 5**.

### 3.2 PFS benefit with the combination of immunotherapy with CCRT

In the single-arm analysis, 45 studies contributed 1−year PFS rate and 38 contributed 2−year PFS rate. Among all evaluated strategies, ICT−CCRT achieved the highest pooled 1−year PFS rate (70.5%, 95% CI 66.2–74.7, I²=58.1%), whereas CCRT-CT had the highest 2−year PFS rate (52.1%, 95% CI 47.7–56.5, I²=58.9%). The PFS rates of immunotherapy−combined regimens were numerically similar to those of chemotherapy−combined strategies, and all combination groups exceeded the rates observed with CCRT alone. Full estimates are provided in **Table 1**. In the pairwise meta-analyses, we included 18 studies. CCRT-IO showed prolonged PFS compared with CCRT (HR 0.74, 95% CI 0.64–0.85, I²=0.0%). No statistically significant differences were observed among the remaining pairwise comparisons. Detailed results are presented in **Figure 2B**.

In the NMA of PFS, 18 studies were included. CCRT-IO was associated with better PFS relative to CCRT alone (HR 0.78, 95% CI 0.76–0.99), consistent with the finding from the pairwise meta-analysis. Additionally, compared with CCRT alone, both ICT-CCRT (HR 0.84, 95% CI 0.71–0.99) and CCRT-CT (HR 0.88, 95% CI 0.79–0.97) demonstrated improved PFS. No significant differences were observed among the remaining treatment strategies (**Figure 3**). The network diagram for PFS is presented in **Figure 4B**. Among the evaluated regimens, ICT-CCRT had the highest probability of ranking first for PFS (0.80), as shown in **Supplementary Figure 4B**.

### 3.3 ORR across treatment strategies

In the single-arm analysis, a total of 31 studies were included with ORR. Among all evaluated strategies, ICT-CCRT yielded the highest pooled ORR (88.4%), followed by CCRT-CT (84.9%). The ORR values of other combination regimens and CCRT alone were relatively lower, ranging from 61.0% to 71.9%. Full data are provided in **Table 1**. Due to limited available data, only CT-CCRT was included in the pairwise meta-analysis. However, no statistically significant difference in ORR was observed compared with CCRT.

### 3.4 Severe adverse events

Data from single−arm studies and the experimental arms of controlled trials were pooled to assess the incidence of severe AEs (grade ≥3), including hematological toxicities, pneumonitis (including radiation-related and immune-related), and esophagitis. The pooled incidence of grade ≥3 AEs was numerically higher in both the ICT-CCRT (56.6%) and CCRT-cIO groups (76.7%) compared with the CCRT alone group (38.9%). This trend was particularly evident for grade ≥3 lymphopenia. The incidence of grade ≥3 anemia was also numerically elevated in the ICT−CCRT group relative to other strategies. In contrast, the rates of grade ≥3 pneumonitis, esophagitis, and other hematological toxicities appeared comparable between the immunotherapy-combined regimens and the CCRT group. However, a formal comparative analysis for these specific toxicities was not feasible due to the limited number of studies reporting them. The summary results are presented in **Table 2**.

**Table 2:** The pooled incidence rates of severe AEs for the 6 treatment regimens in single meta-analysis.

Treatment regimen	NO. of study	NO. of patients	The pooled rate of grade ≥3 overall AEs (95% CI)	NO. of study	NO. of patients	The pooled rate of grade ≥3 pneumonitis (95% CI)	NO. of study	NO. of patients	The pooled rate of grade ≥3 Esophagitis (95% CI)	NO. of study	NO. of patients	The pooled rate of grade ≥3 Leukopenia (95% CI)
ICT-CCRT	7	505	56.6% (36.8-75.4)	12	868	3.0% (1.3-5.2)	12	857	9.6% (6.3-13.5)	9	641	15.6% (9.5-22.8)
CCRT-cIO	3	101	76.7% (50.7-95.3)	8	422	3.0% (0.2-7.7)	6	363	7.9% (1.2-18.4)	6	366	22.8% (12.9-34.3)
CCRT-IO	1	504	13.7% (10.8-16.9)	3	80	0.9% (0.0-5.6)	–	–	–	–	–	–
CT-CCRT	–	–	–	10	1000	3.3% (1.5-5.7)	10	1000	6.0% (3.0-9.9)	6	467	22.7% (11.2-34.3)
CCRT-CT	–	–	–	6	723	1.9% (0.4-4.0)	5	653	10.9% (1.5-26.6)	3	499	45.7% (30.1-61.7)
CCRT	3	377	38.9% (23.7-55.2)	12	1601	2.7% (1.3-4.6)	10	1476	8.1% (4.8-11.4)	7	855	21.9% (11.9-31.8)

Treatment regimen	NO. of study	NO. of patients	The pooled rate of grade ≥3 Neutropenia (95% CI)	NO. of study	NO. of patients	The pooled rate of grade ≥3 Lymphopenia (95% CI)	NO. of study	NO. of patients	The pooled rate of grade ≥3 Anemia (95% CI)	NO. of study	NO. of patients	The pooled rate of grade ≥3 Thrombocytopenia (95% CI)
ICT-CCRT	7	483	14.1% (7.9-21.8)	5	402	79.4% (62.6-92.3)	8	587	11.2% (4.2-20.7)	7	473	6.6% (-2.2-12.7)
CCRT-cIO	6	386	18.9% (7.4-33.7)	3	246	74.9% (56.4-89.7)	5	309	3.9% (1.2-7.7)	5	307	4.1% (-1.9-7.0)
CCRT-IO	–	–	–	–	–	–	–	–	–	–	–	–
CT-CCRT	6	457	14.9% (6.0-26.7)	–	–	–	6	467	0.9% (0.0-1.8)	8	542	8.3% (3.5-14.7)
CCRT-CT	3	499	29.8% (8.3-57.3)	–	–	–	3	461	2.5% (1.1-4.5)	4	569	8.2% (2.6-16.3)
CCRT	8	908	11.6% (5.0-20.2)	–	–	–	8	922	3.1% (0.2-8.1)	9	972	5.5% (2.6-9.4)

The data from single-arm and the experimental group of controlled trials. CI, confidence interval; AEs, adverse events; CCRT, concurrent chemoradiotherapy; ICT-CCRT, induction immunochemotherapy plus CCRT; CCRT-cIO, CCRT plus concurrent immunotherapy; CCRT-IO, CCRT plus consolidation immunotherapy; CT-CCRT, induction chemotherapy plus CCRT; CCRT-CT, CCRT plus consolidation chemotherapy.

### 3.5 Sensitivity analysis and publication bias

Sensitivity analyses were conducted by sequentially omitting each study to assess the robustness of the pooled estimates. The summary results were largely consistent across most comparisons. One exception was the study by Xie et al. (85) in the CCRT-cIO analysis: the point estimate of this study deviated notably from the CI of the combined effect. Because this study was a database-based analysis rather than a real-world clinical cohort, and its removal restored the consistency of the estimate, we excluded it and performed a reanalysis. The corresponding sensitivity plots are presented in the **Supplementary Figures 6**-**10**. Publication bias was assessed via funnel plots and was not evident in most analyses (**Supplementary Figures 11**, **12**); however, asymmetry was detected for PFS in the ICT-CCRT analysis (P<0.05).

## 4 Discussion

Immunotherapy has demonstrated considerable promise in the neoadjuvant setting for operable EC, yet its role in dCCRT for unresectable disease continues to evolve, with current evidence mainly from retrospective studies and phase II trials. To further evaluate the potential benefit of adding immunotherapy to dCCRT, we conducted this systematic review and meta-analysis. For OS, ICT-CCRT significantly improved OS compared with CCRT alone and ranked first among all evaluated strategies in NMA. CCRT-IO also significantly improved OS in pairwise meta-analyses, but this benefit did not reach statistical significance in the NMA, although a favorable trend was observed. For PFS, the benefit of ICT-CCRT over CCRT alone was not consistently observed across analyses, which may reflect differences in statistical methodology; nevertheless, it ranked first in the NMA, suggesting a potential advantage. In contrast, CCRT-IO consistently improved PFS over CCRT alone in both pairwise and network meta-analyses, although its PFS rates were numerically comparable to those of other regimens in single-arm studies. Collectively, adding immunotherapy to dCCRT improves survival outcomes. ICT-CCRT appears most promising for OS and also holds potential for PFS. CCRT-IO is effective for PFS and shows OS benefit only in pairwise comparisons.

Theoretically, the addition of immunotherapy to CCRT could improve treatment effectiveness. This may be attributed to ICIs regulating the tumor microenvironment: PD-1/PD-L1 inhibitors can promote the infiltration of cytotoxic T cells (CD8^+^) and enhance T cell recognition of tumor antigens by inhibiting immunosuppressive signals. Additionally, Tian et al. proposed in *Nature* that PD-1 inhibitors might enhance vascular normalization by activating CD4+ T lymphocytes, thereby overcoming hypoxia, which might improve radiosensitivity (91). Immunotherapy and chemoradiotherapy may synergize to enhance anti-tumor immune responses via the “*in situ* vaccine effect” (radiotherapy promotes the release of tumor antigens) and chemotherapy-induced immunogenic cell death (92). In recent years, neoadjuvant immunotherapy, such as PD-1 inhibitors combined with chemotherapy or CCRT, has shown promising pathological complete response rates in multiple clinical trials (8–11). These findings are consistent with our analysis showing that induction immunochemotherapy improves the prognosis of patients with unresectable EC.

In paired and network meta-analyses, both ICT-CCRT and CCRT-IO demonstrated survival benefits, whereas the CCRT-cIO group did not show a significant advantage. This finding is consistent with evidence from clinical trials such as PACIFIC-2, which demonstrated that the combination of immunotherapy and CCRT does not improve clinical benefit in non-small cell lung cancer (93). The reasons for this failure may involve multiple interrelated factors. First, the most significant challenge is toxicity accumulation: the use of a concurrent regimen substantially increases the risk of high-grade adverse events, leading to premature treatment discontinuation in a considerable proportion of patients due to poor tolerability, thereby preventing completion of the intended immunotherapy course and compromising long-term therapeutic efficacy. In our study, the incidence of severe AEs was higher in the CCRT-cIO group compared with the CCRT alone group (76.7% vs. 38.9%). Second, the timing of drug administration may be suboptimal. Radiation-induced lymphocyte depletion creates an immunosuppressive microenvironment early during CCRT, hindering effective ICIs activation and limiting the expected synergistic effect. Third, the lack of well-defined criteria for identifying optimal patient populations may also contribute to the overall reduction in treatment benefit. In the study by Park et al., it was demonstrated that PD-L1-positive patients exhibited significantly better PFS and OS compared to the PD-L1–negative patients (HR = 0.20, 95% CI 0.07-0.54, P < 0.001; HR = 0.16, 95% CI 0.05-0.56, P = 0.001) (59).

Due to high heterogeneity in analyses of immunotherapy combined with CCRT, we performed subgroup analyses where sample sizes permitted. In the single-arm analysis of ICT-CCRT, stratification by research type showed that prospective studies had lower heterogeneity and higher pooled OS rates than retrospective studies. The high heterogeneity in retrospective studies likely arose from baseline differences, non-standardized treatment, inconsistent data collection, and selection bias. In pairwise meta-analyses, some comparisons also showed considerable heterogeneity, but subgroup analyses were precluded by the limited number of studies. Notably, one study of ICT-CCRT demonstrated that patients who did not receive maintenance immunotherapy after induction immunochemotherapy had significant survival benefits, whereas those who received maintenance therapy showed no additional benefit. Furthermore, the two studies in the CCRT-cIO group differed markedly in design and specific regimens, warranting cautious interpretation of the pooled results. We also identified an induction immunotherapy-monotherapy study (Xu et al., NCT04084158), which reported that adding induction toripalimab and consolidation immunotherapy to CCRT plus consolidation chemotherapy did not significantly improve efficacy in patients with locally advanced ESCC (94). This regimen differed substantially from other induction immunochemotherapy studies; thus, it was excluded from the primary analysis, though its findings merit further consideration. In summary, the optimal combination pattern and timing of radiotherapy with immunotherapy require further investigation. Randomized phase III trials are needed to determine the optimal timing of immunotherapy integration. Future strategies may move beyond a single induction, concurrent, or consolidation approach toward biomarker-driven personalized sequencing of therapies. Several prospective studies of immunotherapy in combination with CCRT are underway and will provide new leads for the optimal treatment pattern for patients with EC (95–99).

Although theoretical considerations suggest that the addition of induction chemotherapy before CCRT may have the potential advantage of reducing tumor stage and shrinking tumor volume, a comprehensive synthesis of both direct and indirect evidence in our study failed to demonstrate a definitive survival benefit associated with this approach. Subgroup analysis by pathological type showed that CT-CCRT improved OS in studies predominantly including squamous cell carcinoma, but not in the adenocarcinoma−predominant studies. These findings suggest that pathological type may influence the efficacy of induction chemotherapy, with potential benefit limited to squamous cell pathological type. Our overall results are consistent with existing evidence. For example, a meta-analysis by Wang J. et al. reported that the addition of induction chemotherapy or consolidation chemotherapy before CCRT improved short-term survival in patients with unresectable EC; however, the long-term survival benefit was not clear (100). Moreover, several studies have failed to demonstrate a significant survival advantage for CT-CCRT (64, 65). In summary, current evidence indicates that CT-CCRT is not an effective strategy to improve prognosis in unselected patients with unresectable EC; however, subgroup analyses suggest a potential benefit in those with squamous cell carcinoma, warranting further validation.

In our analysis, consolidation chemotherapy significantly improved OS, whereas its PFS benefit was only potential. Subgroup analysis of CCRT-CT versus CCRT alone showed that intravenous consolidation chemotherapy improved OS, whereas data for oral regimens were limited to a single study that reported no benefit. The overall OS benefit of consolidation chemotherapy is consistent with a previous meta−analysis by Xia et al. (101). Importantly, our study extends the evidence by including more recent studies and employing single−arm, pairwise, and network meta−analyses, providing robust results that support the efficacy of consolidation chemotherapy.

In the present study, given the limited availability of direct comparative data, we pooled evidence from single-arm trials and experimental arms to synthesize the incidence of AEs across treatment strategies. The addition of immunotherapy to CCRT seems to increase the risk of grade ≥3 AEs overall, particularly lymphopenia and anemia, while the rates of pneumonitis, esophagitis, and other hematological toxicities remained comparable to those with conventional therapy. Nevertheless, these cross-trial comparisons preclude definitive statistical conclusions, as the elevated hematological toxicity may reflect either a genuine synergistic effect or inherent bias in the pooled data. The comparable incidence rates of severe pneumonitis and esophagitis across groups partially alleviate concerns regarding potential pulmonary or esophageal mucosal injury associated with the combination of immunotherapy and thoracic radiotherapy. However, randomized trials with rigorous monitoring are required to determine whether the numerical increases in AEs represent a true safety signal or are due to the limitations of aggregated single-arm data.

In this study, we conducted single-group, pairwise, and network meta-analyses to comprehensively evaluate the efficacy of various combination strategies in addition to CCRT. By applying multiple meta−analytic techniques to data from both controlled trials and a large set of single−arm studies, we were able to cross−validate findings and synthesize all available evidence. This approach enabled a more comprehensive comparison among treatments for the same outcomes and helped identify the most effective therapeutic strategy. To our knowledge, this is the first meta-analysis to evaluate the efficacy and safety of immunotherapy in patients with unresectable EC treated with CCRT. Our analysis further examined the impact of different immunotherapy modes—induction immunochemotherapy, concurrent immunotherapy, and consolidation immunotherapy—on survival outcomes, providing clinicians with valuable insights for treatment selection.

Our meta-analysis had several limitations. First, of the 33 studies included in the NMA, 28 (85%) were retrospective studies. To date, only two randomized controlled trials of immunotherapy with CCRT have been published. The predominance of retrospective studies may affect result interpretation through multiple mechanisms: selection bias (treatment assignment influenced by clinical factors rather than randomization, compromising comparability); unmeasured confounding (e.g., tumor burden, nutritional status, cycles of therapy) that cannot be adjusted for and may either inflate or obscure true treatment effects; and clinical heterogeneity arising from variations in radiotherapy techniques, dose fractionation, and supportive care across centers. Although we used random effects models and subgroup analyses to mitigate these influences, the inherent bias of retrospective data cannot be fully eliminated. Second, data sparsity directly compromises the certainty of conclusions and the power of the NMA. The limited number of controlled trials involving immunotherapy (three for ICT-CCRT and CCRT-IO, two for CCRT-cIO) results in wide CIs around effect estimates and insufficient statistical precision. More importantly, owing to the lack of head-to-head comparisons among several treatment strategies, a closed loop could not be formed for all strategies in the NMA, precluding assessment of local inconsistency (e.g., differences between direct and indirect comparisons). This fundamentally undermines the reliability and statistical power of the NMA for comparing treatment effects. Furthermore, the inconsistent results observed for PFS in ICT-CCRT and for OS in CCRT-IO between pairwise and network meta-analyses further reflect the impact of data sparsity on result stability. To mitigate this issue, we cross-validated findings using single arm, pairwise, and network meta-analyses, but caution remains warranted. Third, some studies did not directly provide HRs; we estimated them from survival curves using the method of Tierney et al. (19), which may introduce approximation errors. Fourth, statistical heterogeneity was detected in some analyses; we performed subgroup analyses stratified by research type, pathological type, and administration route to contextualize observed differences and used random effects models to conservatively account for heterogeneity. Sensitivity analyses confirmed the general stability of the pooled estimates, with the exception of one study that was excluded due to methodological divergence. Publication bias was not evident across the majority of outcomes; however, funnel plot asymmetry was observed for PFS in the ICT-CCRT analysis (P <0.05), suggesting potential selective reporting. Given the limited number of studies included, this finding should be interpreted with caution. In summary, our conclusions should be generalized cautiously within the context of the available evidence.

In conclusion, compared with CCRT alone, ICT−CCRT significantly improves OS and shows a potential trend toward in PFS benefit; in the NMA, ICT-CCRT ranked as the most favorable among all evaluated strategies, suggesting it may be the most effective regimen. CCRT−IO improves OS only in pairwise meta−analyses, but consistently improves PFS compared with CCRT alone in the pairwise and network meta-analysis, although its pooled PFS rates from single−arm studies are numerically comparable to those of other regimens. Currently, sufficient randomized controlled trials are lacking to validate the efficacy of immunotherapy combined with CCRT and to determine the optimal timing and sequence of their integration. Future well−designed prospective studies are needed to guide the optimization of personalized treatment strategies.

## Data Availability

The original contributions presented in the study are included in the article/**Supplementary Material**. Further inquiries can be directed to the corresponding authors.
