# Supplementary material for: Induction immunochemotherapy followed by concurrent chemoradiotherapy improves survival in unresectable esophageal cancer: a systematic review, meta-analysis, and network meta-analysis
Source: Front Immunol. 2026 May 12;17:1767380. doi: 10.3389/fimmu.2026.1767380 (PMC13201479; doi:10.3389/fimmu.2026.1767380)
Supplement: Supplementary file 1 [file Image1.pdf]

Supplementary material

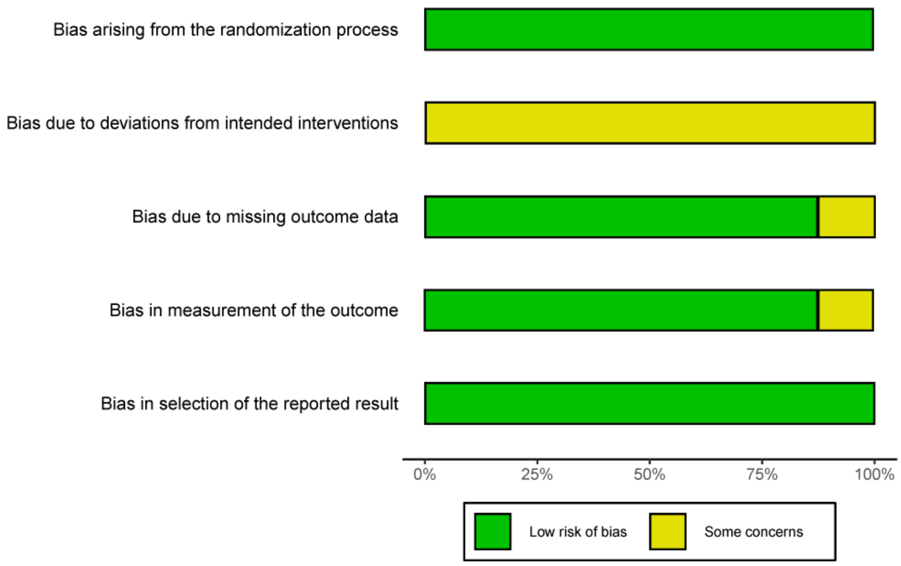

Supplementary Figure 1. Risk of bias and quality assessment for RCTs on summary

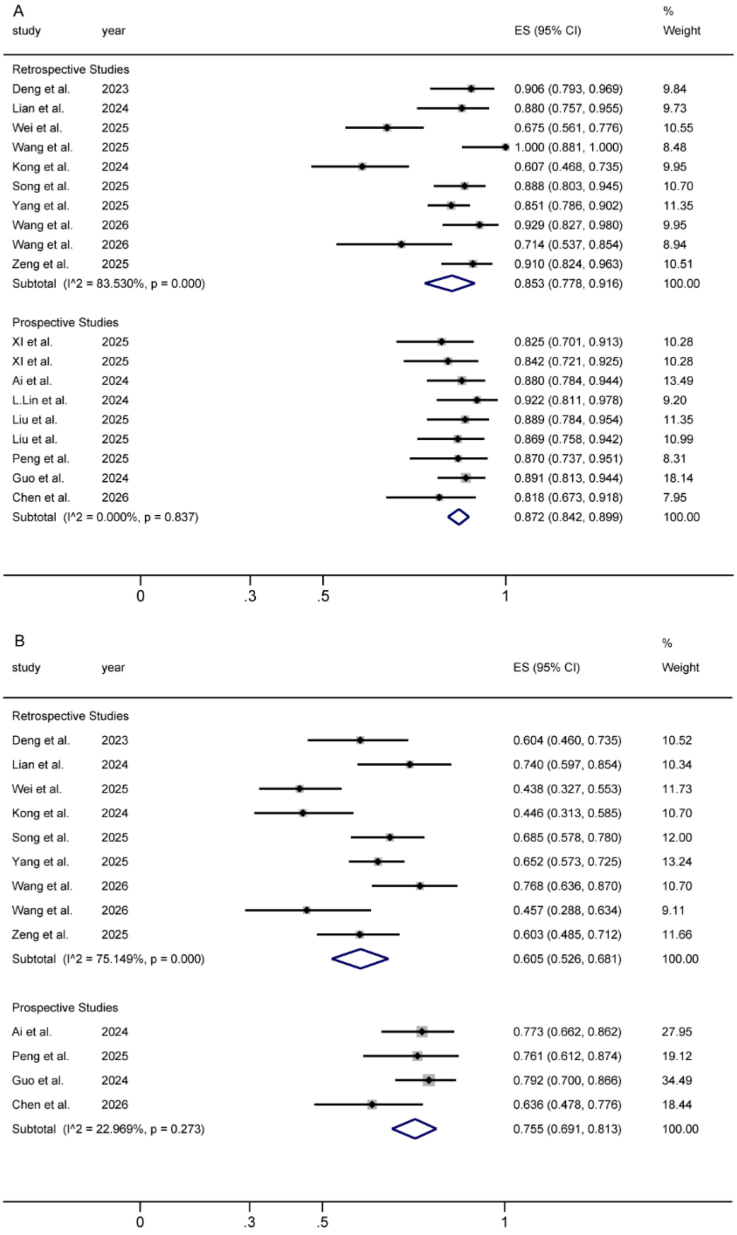

Supplementary Figure 2. Subgroup analysis of 1-year and 2-year OS rates in the ICT-CCRT group by research type

Forest plots showing subgroup analyses of pooled 1-year (A) and 2-year (B) OS rates for the ICT-CCRT regimen, stratified by research design (prospective versus retrospective). Pooled estimates were calculated using random-effects models. CCRT=concurrent chemoradiotherapy. ICT-CCRT= induction immunochemotherapy plus CCRT.

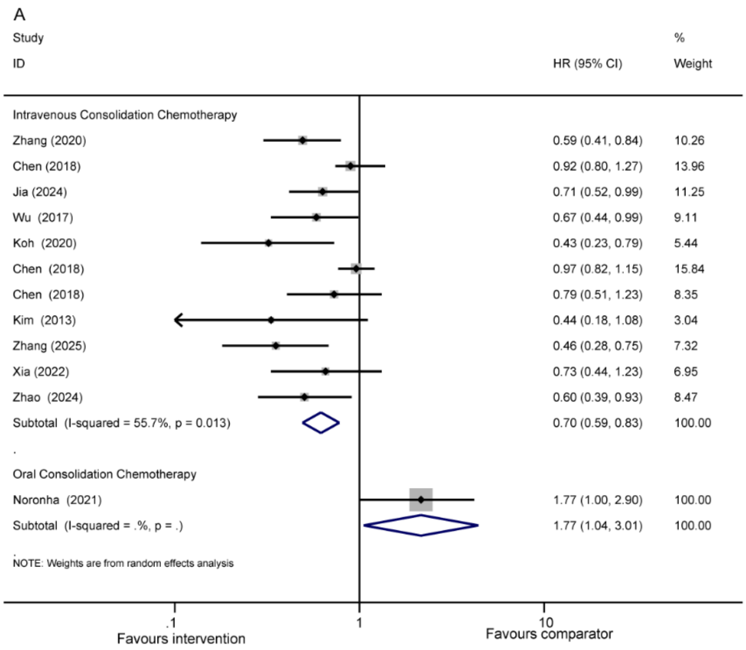

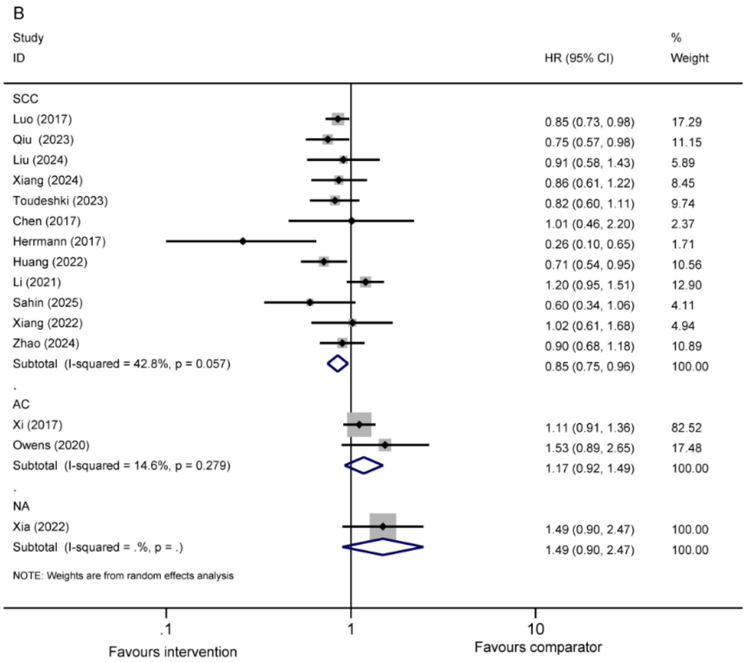

Supplementary Figure 3. Subgroup analyses of OS for CCRT-CT versus CCRT alone, and CT-CCRT versus CCRT alone

(A)Comparison of CCRT-CT versus CCRT alone, stratified by route of consolidation chemotherapy administration (intravenous versus oral). (B) Comparison of CT-CCRT versus CCRT alone, stratified by predominant pathological type (SCC versus AC). HRs with 95% CIs were calculated using random-effects models. SCC=squamous cell carcinoma. AC=adenocarcinoma. HRs=Hazard ratios. CIs=confidence intervals. CCRT=concurrent chemoradiotherapy. CT-CCRT= induction chemotherapy plus CCRT. CCRT-CT= CCRT plus consolidation chemotherapy.

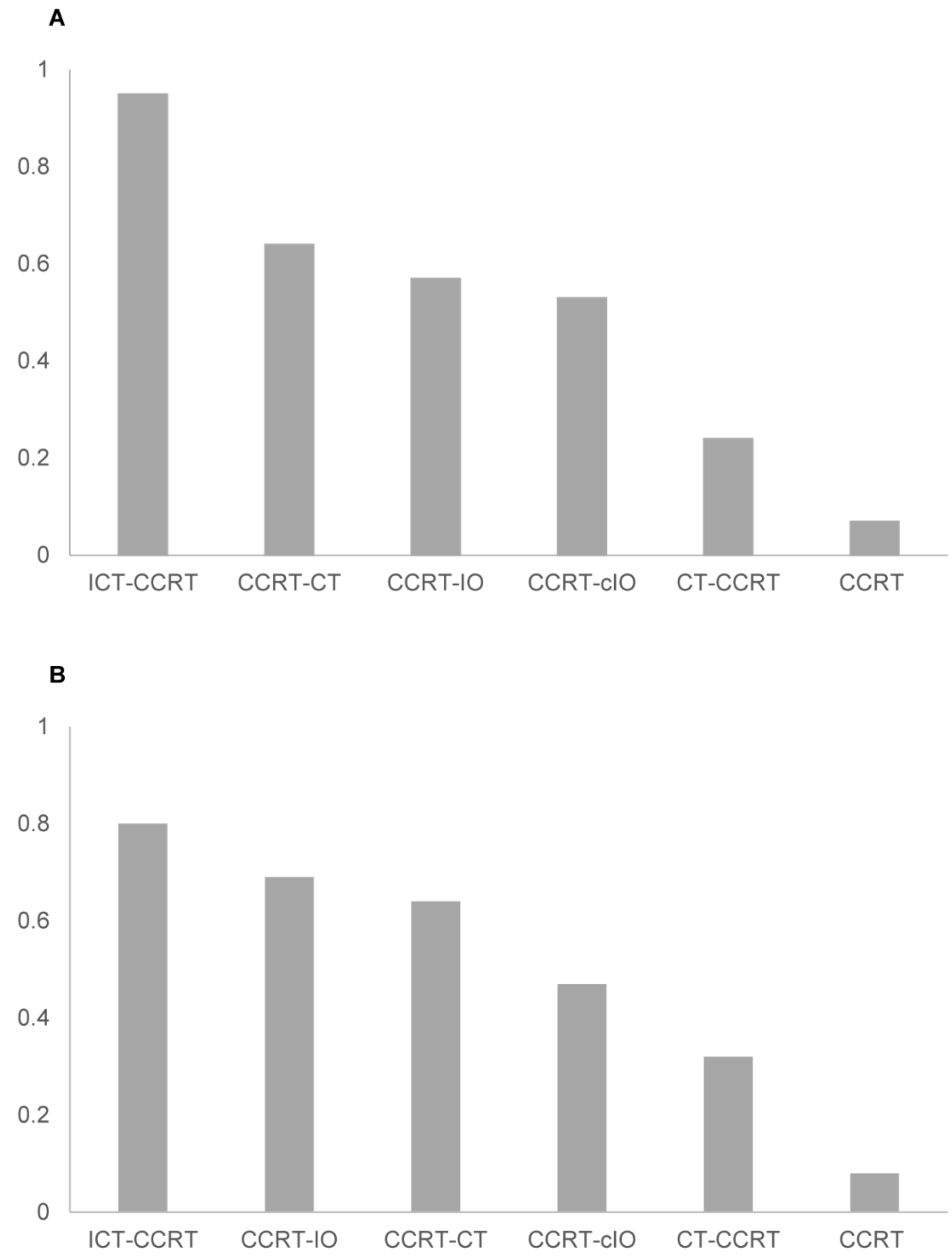

Supplementary Figure 4. Ranking probabilities for the (A)OS and (B)PFS

CCRT=concurrent chemoradiotherapy. ICT-CCRT= induction immunochemotherapy plus CCRT. CCRT-IO=CCRT plus consolidation immunotherapy. CCRT-cIO= CCRT plus concurrent immunotherapy. CT-CCRT= induction chemotherapy plus CCRT. CCRT-CT= CCRT plus consolidation chemotherapy.

A

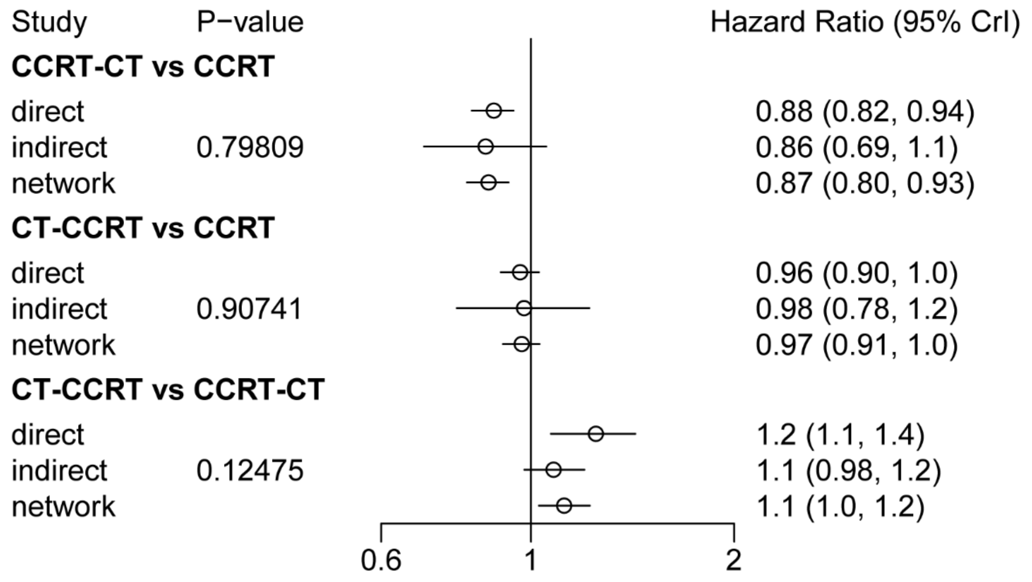

B

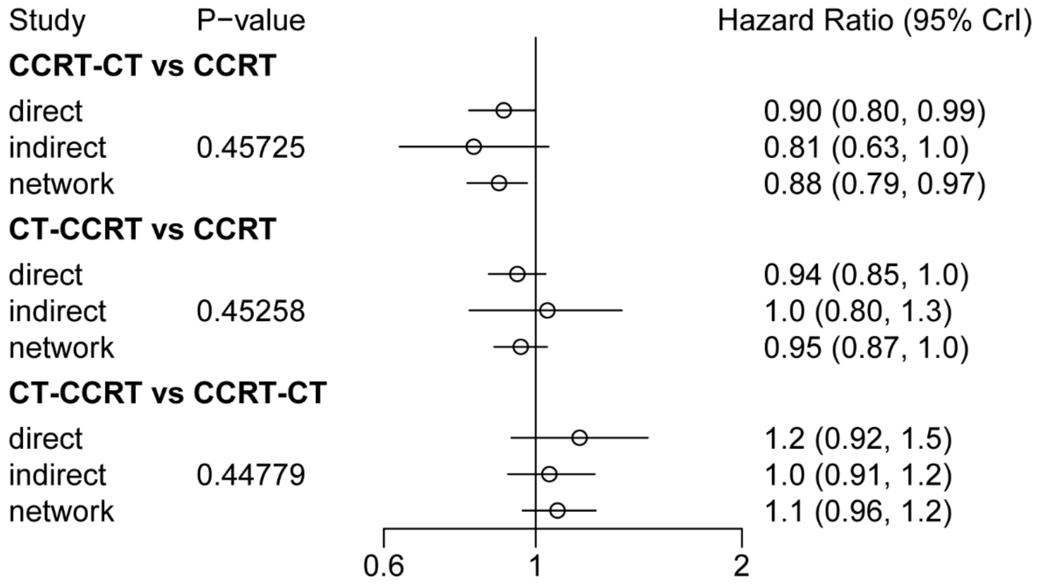

Supplementary Figure 5. Assessment of local inconsistency using the node-splitting method for CCT-CT, CT-CCRT and CCRT in (A)OS and (B)PFS

The table presents the HRs with 95% CrIs derived from direct evidence, indirect evidence, and the combined network meta-analysis estimate for each comparison. The P-value indicates the significance of the difference between the direct and indirect estimates. A $P > 0.05$ suggests no significant inconsistency between the direct and indirect evidence for that particular comparison. HR=hazard ratio. CCRT=concurrent chemoradiotherapy. CT-CCRT= induction chemotherapy plus CCRT. CCRT-CT= CCRT plus consolidation chemotherapy.

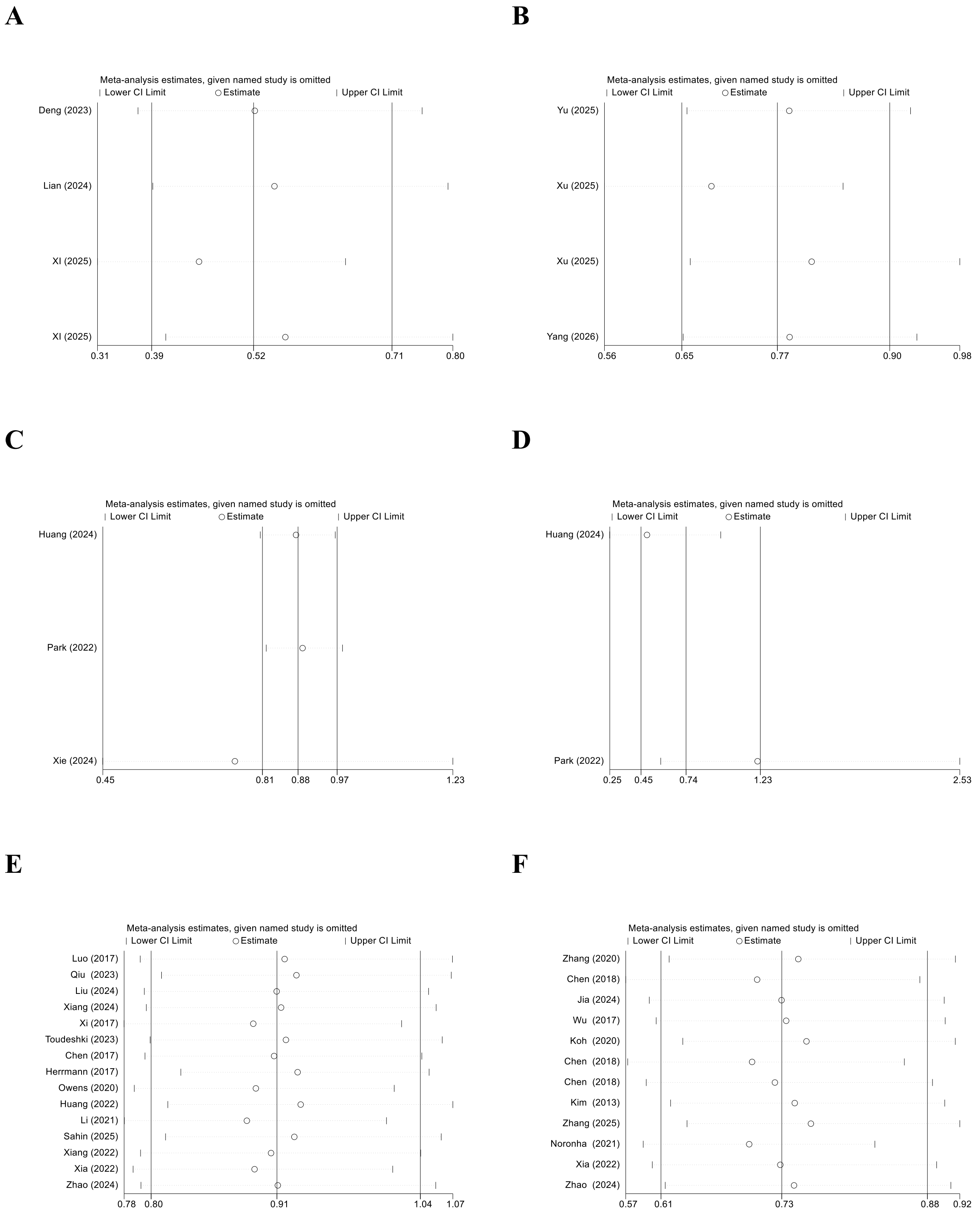
A
Meta-analysis estimates, given named study is omitted
Lower CI Limit
Estimate
Upper CI Limit
Deng (2023)
Lian (2024)
XI (2025)
XI (2025)
0.31 0.39 0.52 0.71 0.80
B
Meta-analysis estimates, given named study is omitted
Lower CI Limit
Estimate
Upper CI Limit
Yu (2025)
Xu (2025)
Xu (2025)
Yang (2026)
0.56 0.65 0.77 0.90 0.98
C
Meta-analysis estimates, given named study is omitted
Lower CI Limit
Estimate
Upper CI Limit
Huang (2024)
Park (2022)
Xie (2024)
0.45 0.81 0.88 0.97 1.23
D
Meta-analysis estimates, given named study is omitted
Lower CI Limit
Estimate
Upper CI Limit
Huang (2024)
Park (2022)
0.25 0.45 0.74 1.23 2.53
E
Meta-analysis estimates, given named study is omitted
Lower CI Limit
Estimate
Upper CI Limit
Luo (2017)
Qiu (2023)
Liu (2024)
Xiang (2024)
Xi (2017)
Toudeshki (2023)
Chen (2017)
Herrmann (2017)
Owens (2020)
Huang (2022)
Li (2021)
Sahin (2025)
Xiang (2022)
Xia (2022)
Zhao (2024)
0.78 0.80 0.91 1.04 1.07
F
Meta-analysis estimates, given named study is omitted
Lower CI Limit
Estimate
Upper CI Limit
Zhang (2020)
Chen (2018)
Jia (2024)
Wu (2017)
Koh (2020)
Chen (2018)
Chen (2018)
Kim (2013)
Zhang (2025)
Noronha (2021)
Xia (2022)
Zhao (2024)
0.57 0.61 0.73 0.88 0.92

G

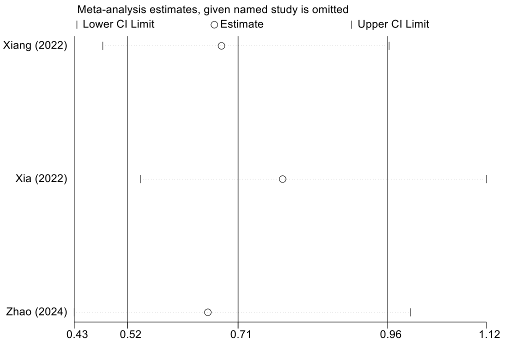

Supplementary Figure 6. Sensitivity analysis of HRs of OS

Sensitivity analyses of the outcomes by repeating the pooled analyses with one study omitted at a time. (A)ICT-CCRT vs CCRT. (B)CCRT-IO vs CCRT. (C)CCRT-cIO vs CCRT. (D) CCRT-cIO vs CCRT after excluding the study by Xie et al. (E)CT-CCRT vs CCRT. (F) CCRT-CT vs CCRT. (G)CCRT-CT vs CT-CCRT. HR=hazard ratio. CCRT=concurrent chemoradiotherapy. ICT-CCRT= induction immunochemotherapy plus CCRT. CCRT-IO=CCRT plus consolidation immunotherapy. CCRT-cIO= CCRT plus concurrent immunotherapy. CT-CCRT= induction chemotherapy plus CCRT. CCRT-CT= CCRT plus consolidation chemotherapy.

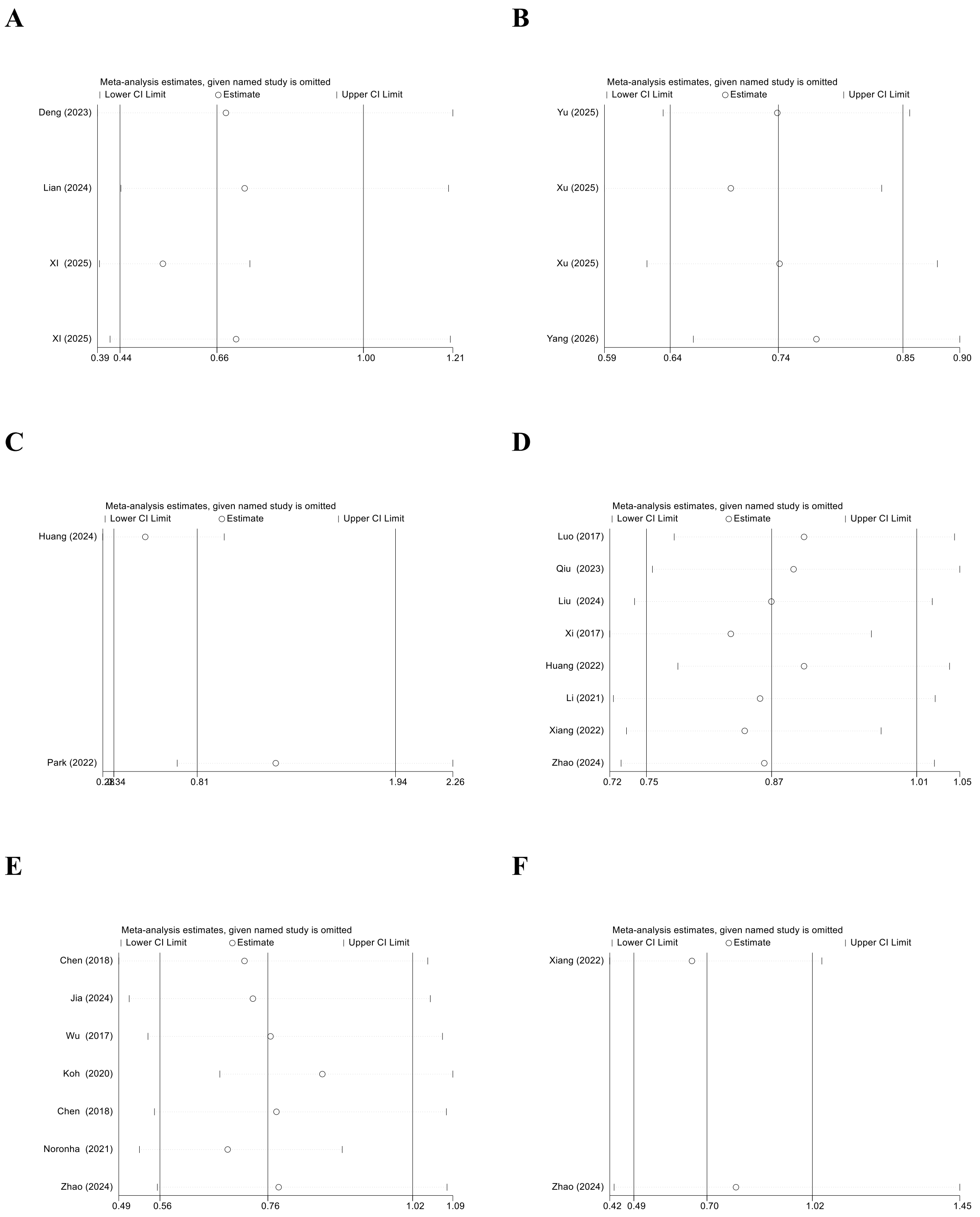

Supplementary Figure 7. Sensitivity analysis of HRs of PFS

Sensitivity analyses of the outcomes by repeating the pooled analyses with one study omitted at a time. (A)ICT-CCRT vs CCRT. (B)CCRT-IO vs CCRT. (C)CCRT-cIO vs CCRT. (D) CT-CCRT vs CCRT. (E) CCRT-CT vs CCRT. (F)CCRT-CT vs CT-CCRT. HR=hazard ratio. CCRT=concurrent chemoradiotherapy. ICT-CCRT= induction immunochemotherapy plus CCRT. CCRT-IO=CCRT plus consolidation immunotherapy. CCRT-cIO= CCRT plus concurrent immunotherapy. CT-CCRT= induction chemotherapy plus CCRT. CCRT-CT= CCRT plus consolidation chemotherapy.

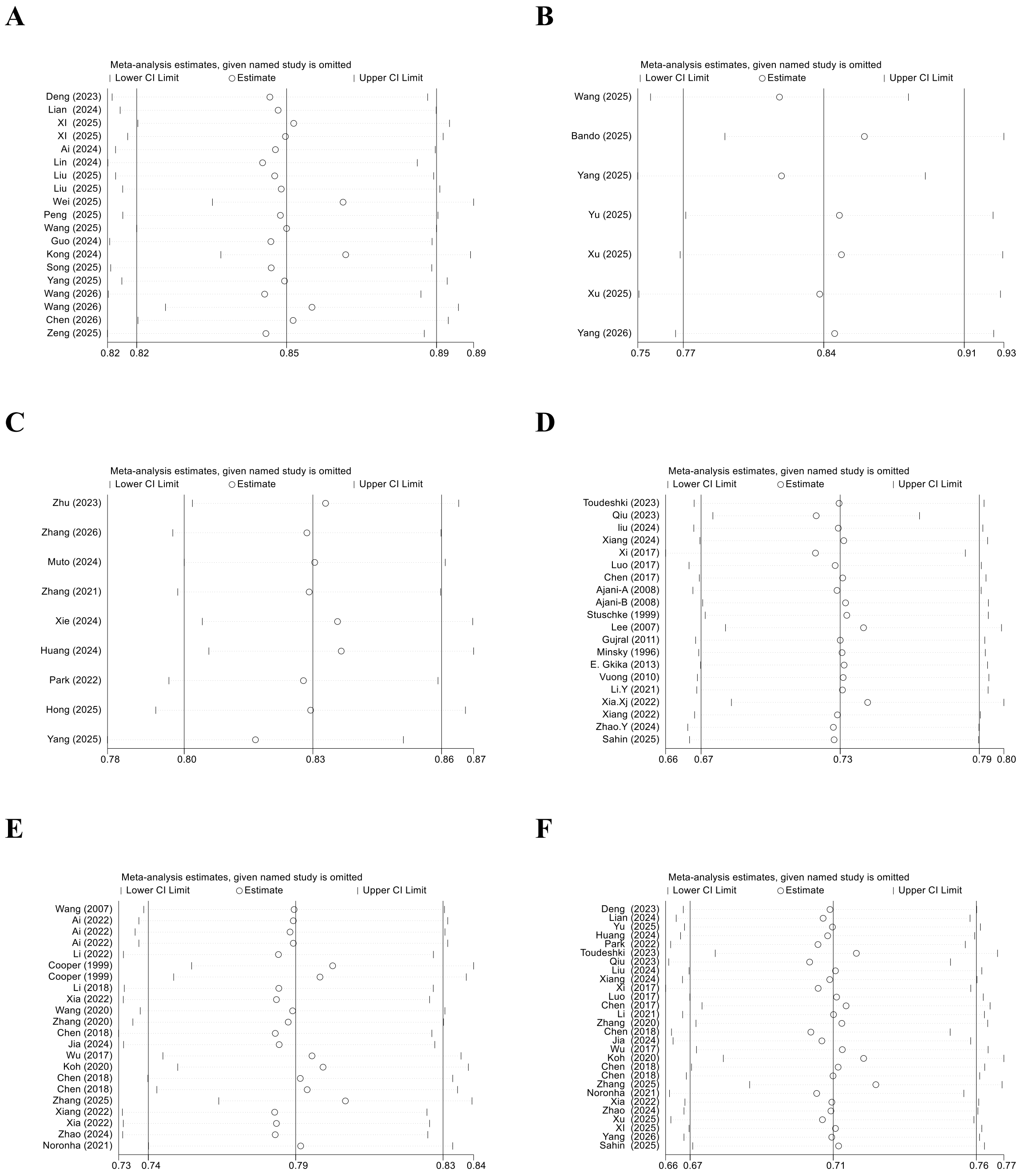

Supplementary Figure 8. Sensitivity analysis of the pooled 1-year OS rate (single-arm meta-analysis)

Sensitivity analyses of the outcomes by repeating the pooled analyses with one study omitted at a time. (A)ICT-CCRT (B)CCRT-IO (C)CCRT-cIO (D) CT-CCRT (E) CCRT-CT (F)CCRT. CCRT=concurrent chemoradiotherapy. ICT-CCRT= induction immunochemotherapy plus CCRT. CCRT-cIO= CCRT plus concurrent immunotherapy. CCRT-IO=CCRT plus consolidation immunotherapy. CT-CCRT= induction chemotherapy plus CCRT. CCRT-CT= CCRT plus consolidation chemotherapy.

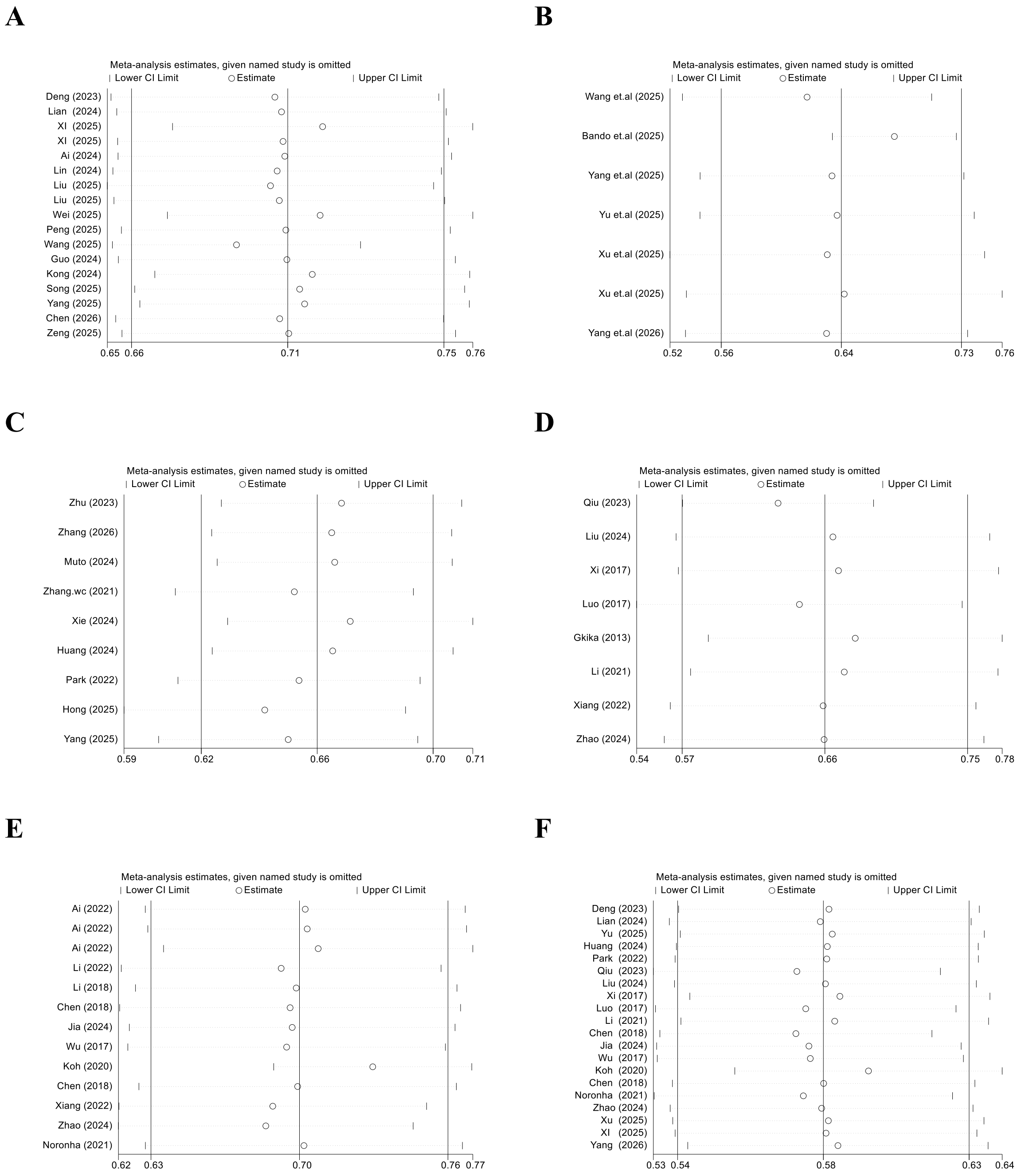

Supplementary Figure 9. Sensitivity analysis of the pooled 1-year PFS rate (single-arm meta-analysis)

Sensitivity analyses of the outcomes by repeating the pooled analyses with one study omitted at a time. (A)ICT-CCRT (B)CCRT-IO (C)CCRT-cIO (D) CT-CCRT (E) CCRT-CT (F)CCRT. CCRT=concurrent chemoradiotherapy. ICT-CCRT= induction immunochemotherapy plus CCRT. CCRT-IO=CCRT plus consolidation immunotherapy. CCRT-cIO= CCRT plus concurrent immunotherapy. CT-CCRT= induction chemotherapy plus CCRT. CCRT-CT= CCRT plus consolidation chemotherapy.

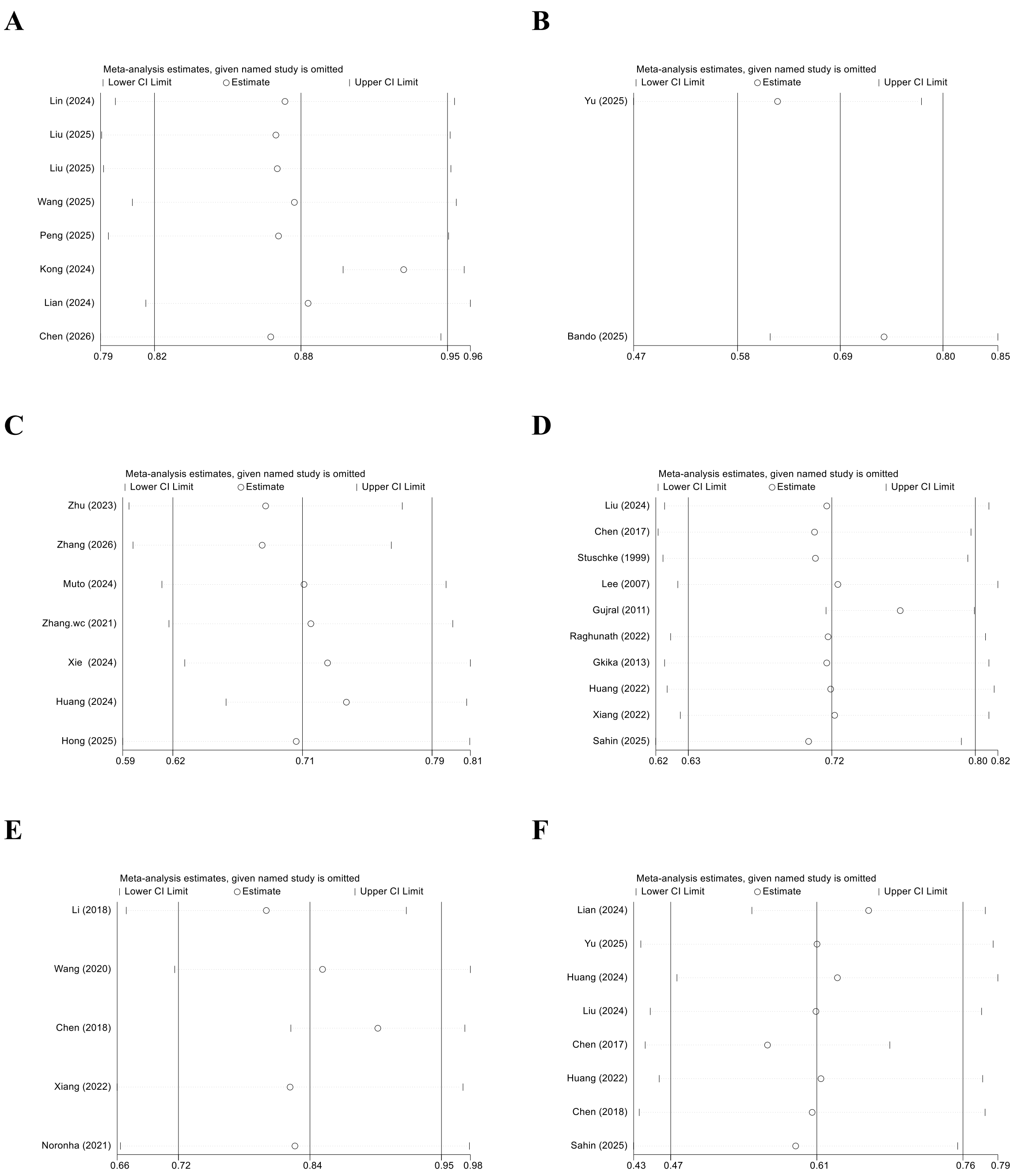

Supplementary Figure 10. Sensitivity analysis of the pooled ORR rate (single-arm meta-analysis)

Sensitivity analyses of the outcomes by repeating the pooled analyses with one study omitted at a time. (A)ICT-CCRT (B)CCRT-IO (C)CCRT-cIO (D) CT-CCRT (E) CCRT-CT (F)CCRT. CCRT=concurrent chemoradiotherapy. ICT-CCRT= induction immunochemotherapy plus CCRT. CCRT-IO=CCRT plus consolidation immunotherapy. CCRT-cIO= CCRT plus concurrent immunotherapy. CT-CCRT= induction chemotherapy plus CCRT. CCRT-CT= CCRT plus consolidation chemotherapy.

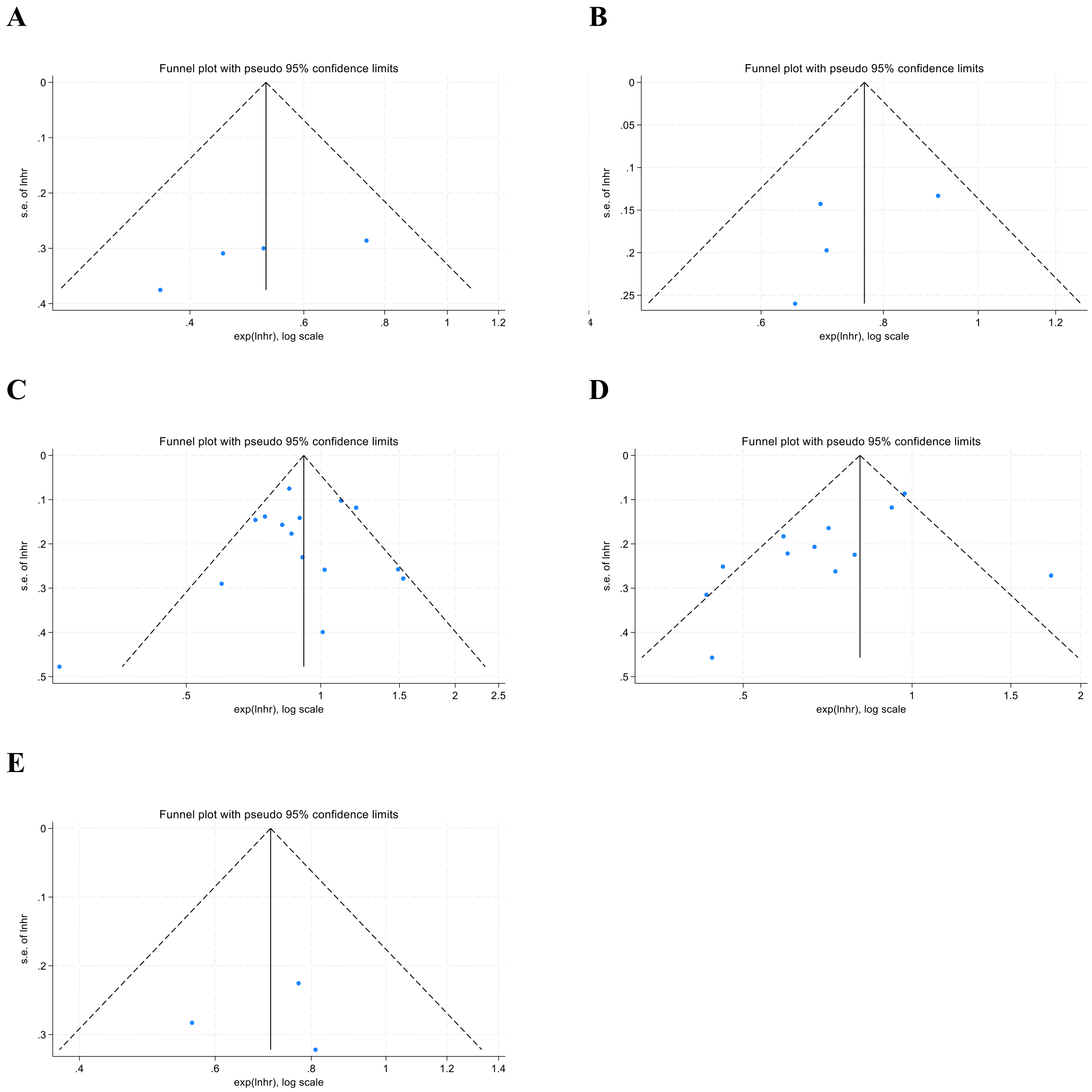

Supplementary Figure 11. Funnel plot of publication bias for OS (pairwise meta-analysis)

(A)ICT-CCRT vs CCRT. (B)CCRT-IO vs CCRT. (C)CT-CCRT vs CCRT. (D) CCRT-CT vs CCRT. (E)CCRT-CT vs CT-CCRT. CCRT=concurrent chemoradiotherapy. ICT-CCRT= induction immunochemotherapy plus CCRT. CCRT-IO=CCRT plus consolidation immunotherapy. CCRT-cIO= CCRT plus concurrent immunotherapy. CT-CCRT= induction chemotherapy plus CCRT. CCRT-CT= CCRT plus consolidation chemotherapy.

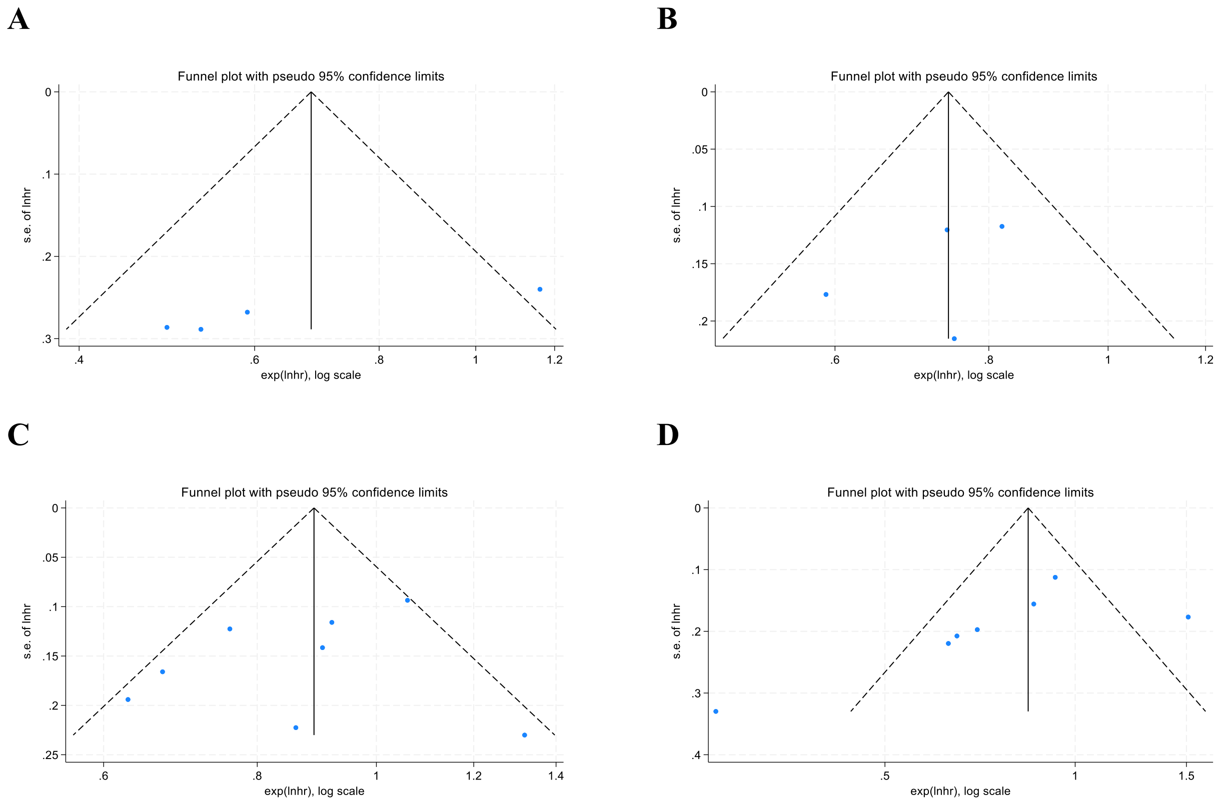

Supplementary Figure 12. Funnel plot of publication bias for PFS (pairwise meta-analysis)

(A)ICT-CCRT vs CCRT. (B)CCRT-IO vs CCRT. (C)CT-CCRT vs CCRT. (D) CCRT-CT vs CCRT. CCRT=concurrent chemoradiotherapy. ICT-CCRT= induction immunochemotherapy plus CCRT. CCRT-IO=CCRT plus consolidation immunotherapy. CCRT-cIO= CCRT plus concurrent immunotherapy. CT-CCRT= induction chemotherapy plus CCRT. CCRT-CT= CCRT plus consolidation chemotherapy.
