# Supplementary material for: Induction immunochemotherapy followed by concurrent chemoradiotherapy improves survival in unresectable esophageal cancer: a systematic review, meta-analysis, and network meta-analysis
Source: Front Immunol. 2026 May 12;17:1767380. doi: 10.3389/fimmu.2026.1767380 (PMC13201479; doi:10.3389/fimmu.2026.1767380)
Supplement: Supplementary file 2 [file Table1.docx]

**Supplementary material**

**Supplementary Table 1 Search Strategies**

PubMed

((esophageal squamous cell carcinoma[Title/Abstract]) OR (Esophageal Neoplasm[Title/Abstract]) OR (Esophagus Cancer[Title/Abstract]) OR (Esophageal Cancer[Title/Abstract]) OR ("Esophageal Neoplasms"[Mesh])) AND (("Chemoradiotherapy"[Mesh]) OR ("Radiotherapy"[Mesh]) OR (chemoradiotherapy[Title/Abstract]) OR (chemoradiation therapy[Title/Abstract]) OR (chemoradiation[Title/Abstract]) OR (radiotherapy[Title/Abstract])) AND ((consolidation[Title/Abstract]) OR (Neoadjuvant[Title/Abstract]) OR (Induction[Title/Abstract]) OR ("Consolidation Chemotherapy"[Mesh]) OR ("Neoadjuvant Therapy"[Mesh]) OR ("Induction Chemotherapy"[Mesh]) OR (immunotherapy[Title/Abstract]) OR (immune checkpoint inhibitors[Title/Abstract]) OR (PD-L1 Inhibitors[Title/Abstract]) OR (PD 1 Inhibitors[Title/Abstract]) OR (CTLA-4 Inhibitors[Title/Abstract]) OR ("Immune Checkpoint Inhibitors"[Mesh]) OR ("Immunotherapy"[Mesh])) AND ((locally advanced[Title/Abstract]) OR (unresectable[Title/Abstract]) OR (inoperable[Title/Abstract]) OR (advanced[Title/Abstract]) OR (non-metastatic[Title/Abstract]))

Embase

('esophageal squamous cell carcinoma':ti,ab,kw OR 'esophagus carcinoma':ti,ab,kw OR 'esophagus tumor':ti,ab,kw OR 'esophageal neoplasm':ti,ab,kw OR 'esophageal cancer':ti,ab,kw OR 'esophagus cancer':ti,ab,kw OR 'esophagus tumor'/exp OR 'esophagus cancer'/exp OR 'esophageal neoplasms'/exp) AND ('chemoradiotherapy'/exp OR 'radiotherapy'/exp OR chemoradiotherapy:ti,ab,kw OR 'chemoradiation therapy':ti,ab,kw OR chemoradiation:ti,ab,kw OR radiotherapy:ti,ab,kw) AND (consolidation:ti,ab,kw OR neoadjuvant:ti,ab,kw OR induction:ti,ab,kw OR 'consolidation chemotherapy'/exp OR 'neoadjuvant therapy'/exp OR 'induction chemotherapy'/exp OR immunotherapy:ti,ab,kw OR 'immune checkpoint inhibitor':ti,ab,kw OR 'immune checkpoint inhibitors':ti,ab,kw OR 'pd-1 inhibitor':ti,ab,kw OR 'pd-l1 inhibitor':ti,ab,kw OR 'ctla-4 inhibitor':ti,ab,kw OR 'immunotherapy'/exp OR 'immune checkpoint inhibitor'/exp) AND ('locally advanced':ti,ab,kw OR unresectable:ti,ab,kw OR inoperable:ti,ab,kw OR advanced:ti,ab,kw OR 'non-metastatic':ti,ab,kw) AND 'human'/de AND [embase]/lim NOT ([embase]/lim AND [medline]/lim)

Web of science

TS=("esophageal squamous cell carcinoma" OR "esophagus carcinoma" OR "esophagus tumor" OR "esophageal neoplasm" OR "esophageal cancer" OR "esophagus cancer" OR "oesophageal cancer" OR "oesophageal carcinoma") AND TS=("locally advanced" OR unresectable OR inoperable OR advanced OR "non-metastatic") AND TS=(chemoradiotherapy OR "chemoradiation therapy" OR chemoradiation OR radiotherapy) AND TS=(consolidation OR neoadjuvant OR induction OR immunotherapy OR "immune checkpoint inhibitor" OR "immune checkpoint inhibitors" OR "PD-1 inhibitor" OR "PD-L1 inhibitor" OR "CTLA-4 inhibitor")

Cochrane Library

#1 MeSH descriptor: [Esophageal Neoplasms] explode all trees

#2 (esophageal):ti,ab,kw OR (esophagus):ti,ab,kw OR (oesophageal):ti,ab,kw

#3 (cancer):ti,ab,kw OR (carcinoma):ti,ab,kw OR (tumor):ti,ab,kw OR (tumour):ti,ab,kw OR (neoplasm):ti,ab,kw OR (malignancy):ti,ab,kw

#4 #2 AND #3

#5 (“esophageal squamous cell carcinoma”):ti,ab,kw OR (“esophagus carcinoma”):ti,ab,kw OR (“esophagus tumor”):ti,ab,kw OR (“esophageal neoplasm”):ti,ab,kw OR (“esophageal cancer”):ti,ab,kw OR (“esophagus cancer”):ti,ab,kw

#6 #1 OR #4 OR #5

#7 (“locally advanced”):ti,ab,kw OR (unresectable):ti,ab,kw OR (inoperable):ti,ab,kw OR (advanced):ti,ab,kw OR (non-metastatic):ti,ab,kw

#8 MeSH descriptor: [Chemoradiotherapy] explode all trees

#9 MeSH descriptor: [Radiotherapy] explode all trees

#10 (chemoradiotherapy):ti,ab,kw OR (chemoradiation therapy):ti,ab,kw OR (chemoradiation):ti,ab,kw OR (radiotherapy):ti,ab,kw

#11 #8 OR #9 OR #10

#12 (consolidation):ti,ab,kw OR (neoadjuvant):ti,ab,kw OR (induction):ti,ab,kw

#13 MeSH descriptor: [Consolidation Chemotherapy] explode all trees

#14 MeSH descriptor: [Neoadjuvant Therapy] explode all trees

#15 MeSH descriptor: [Induction Chemotherapy] explode all trees

#16 MeSH descriptor: [Immunotherapy] explode all trees

#17 MeSH descriptor: [Immune Checkpoint Inhibitors] explode all trees

#18 (immunotherapy):ti,ab,kw OR (“immune checkpoint inhibitor”):ti,ab,kw OR (“immune checkpoint inhibitors”):ti,ab,kw OR (“PD-1 inhibitor”):ti,ab,kw OR (“PD-L1 inhibitor”):ti,ab,kw OR (“CTLA-4 inhibitor”):ti,ab,kw

#19 #12 OR #13 OR #14 OR #15 OR #16 OR #17

#20 #6 AND #7 AND #11 AND #19

Filter Language: English Source: ICTRP/CINAHL

ClinicalTrials.gov

(esophageal cancer OR esophageal neoplasm OR esophagus cancer OR esophageal squamous cell carcinoma) AND (chemoradiotherapy OR radiotherapy OR chemoradiation) AND (immunotherapy OR immune checkpoint inhibitor OR PD-1 inhibitor OR PD-L1 inhibitor OR CTLA-4 inhibitor OR consolidation OR induction) AND (locally advanced OR unresectable OR inoperable OR advanced) NOT Surgery

**Supplementary Table 3 Comparison of goodness-of-fit between consistency and inconsistency models**

|  |  | DIC | I^2^ |
| --- | --- | --- | --- |
| OS | CM | 67.1 | 14% |
|  | IM | 68.1 | 15% |
| PFS | CM | 50.3 | 16% |
|  | IM | 51.3 | 17% |

Model fit was assessed using the DIC, with lower values indicating better fit. The I² statistic quantified between-study heterogeneity. A DIC difference < 5 between the CM and IM was considered negligible, supporting the consistency assumption. CM=consistency model. IM=inconsistency model. DIC=deviance information criterion.
